# Isoleucine and valine promote prostate cancer progression via propionyl-CoA-mediated cholesterol metabolism

**DOI:** 10.1038/s42255-026-01583-z

**Published:** 2026-08-20

**Authors:** Zhongchi Li, Shuchen Liu, Wenbing Jin, Leyi Xiao, Olivia Kester, Nayah Bullen, Xuanrong Chen, Un In Chan, Jude Owiredu, Zhucui Li, Rabia Khan, Jennifer Endress, Moniquetta Shafer, Vivien Low, Nikolaos Koundouros, Sungyun Cho, Christopher Barbieri, Chun-Jun Guo, John Blenis

**Affiliations:** 1 Sandra and Edward Meyer Cancer Center, Weill Cornell Medicine, New York, NY USA; 2 Department of Pharmacology, Weill Cornell Medicine, New York, NY USA; 3 Jill Roberts Institute for Research in Inflammatory Bowel Disease, GI Division, Department of Medicine, Weill Cornell Medicine, New York, NY USA; 4 Department of Urology, Weill Cornell Medical College, Cornell University, New York, NY USA; 5 Department of Biochemistry, Weill Cornell Medicine, New York, NY USA; 6 Proteomics and Metabolomics Core Facility, Weill Cornell Medicine, New York, NY USA; 7 Cancer Biology & Genetics Program, Memorial Sloan Kettering Cancer Center, New York, NY USA; 8 Department of Pharmacology, Korea University College of Medicine, Korea University, Seoul, South Korea

**Keywords:** Cancer metabolism, Prostate cancer, Metabolism, Post-translational modifications, Sterols

## Abstract

Enhanced cholesterol synthesis and lipid droplet accumulation are hallmarks of aggressive prostate cancer, yet how tumour cells sense metabolic inputs to dynamically regulate cholesterol homeostasis remains poorly defined. Here we uncover a metabolic signalling mechanism in which prostate cancer cells remodel propionyl-CoA metabolism to support stress adaptation during disease progression. We show that the catabolism of branched-chain amino acid, specifically isoleucine and valine, is the primary source of intracellular propionyl-CoA in prostate cancer cells. Beyond its metabolic role, propionyl-CoA functions as a signalling molecule that stabilizes nuclear sterol regulatory element-binding protein 2 (SREBP2) through site-specific lysine propionylation, thereby enhancing its transcriptional activity. This activation promotes cholesterol biosynthesis, fuels de novo androgen production, and sustains androgen receptor signalling under metabolic and therapeutic stress, including androgen deprivation. Together, our findings establish propionyl-CoA as a key metabolic signal linking amino acid catabolism to cholesterol-driven oncogenic programmes and highlight targeting isoleucine and valine metabolism as a potential strategy to disrupt lipid reprogramming in prostate cancer.

## Abstract (web summary)

Rewiring of branched-chain amino acid catabolism in prostate cancer fuels propionyl-CoA synthesis, lysine propionylation and stabilization of SREBP2, leading to increased cholesterol and androgen synthesis, and thus promotes metabolic adaptation of cancer cells and disease progression.

## Main

Prostate cancer (PCa) is the second most commonly diagnosed cancer in men and a leading cause of cancer-related mortality worldwide^1^. While androgen receptor (AR)-targeted therapies initially provide clinical benefit, resistance frequently develops, often leading to castration-resistant prostate cancer (CRPC), a lethal stage of the disease^2^. Mounting evidence indicates that metabolic reprogramming, particularly increased lipid synthesis, storage and utilization, is a hallmark of PCa progression and therapy resistance^3,4^. Targeting metabolic pathways has therefore emerged as a promising therapeutic strategy^5,6^.

Among metabolic factors, cholesterol plays a pivotal role in promoting tumour growth and enabling adaptation to metabolic and therapeutic stress^3,7^. In cancer cells, cholesterol homeostasis is tightly controlled by sterol regulatory element-binding protein 2 (SREBP2), which senses intracellular cholesterol levels and adjusts cholesterol synthesis accordingly^8,9^. Under low-cholesterol conditions, SREBP2 is cleaved, and its active N-terminal fragment translocates to the nucleus to induce cholesterol biosynthesis and uptake genes. When cholesterol is abundant, this cleavage is suppressed via negative feedback, a mechanism that seems to be attenuated in PCa compared with normal prostate tissue^9^. During tumour progression and in response to therapy-induced stress, cholesterol demand remains persistently high^10^. To sustain SREBP2 activity under these conditions, tumour cells must circumvent this canonical feedback inhibition. However, the mechanisms by which cancer cells override this regulatory axis remain poorly understood, representing a critical gap in our understanding of cholesterol metabolism in malignancy.

Mitochondria are increasingly recognized not only as bioenergetic centres but also as key hubs of metabolic signalling^11^. Among mitochondrial metabolites, acyl-CoAs have emerged as regulators of protein function via post-translational modifications^12^. While acetyl-CoA has been widely studied in this context^13^, the role of other acyl-CoAs remains relatively underexplored. Notably, propionyl-CoA (P-CoA), which accumulates in mitochondria and nuclei, has been suggested to serve signalling functions beyond its canonical metabolic role^14^. P-CoA is primarily catabolized via the evolutionarily conserved propionate metabolism pathway. Recent studies, including our own, have shown that this pathway can be reprogrammed in cancer cells, and its dysregulation may lead to the buildup of metabolic intermediates that contribute to tumour progression^15–18^.

In this study, we uncover a previously unrecognized mechanism by which PCa cells exploit P-CoA as a signalling metabolite to activate SREBP2 and drive cholesterol biosynthesis. We show that P-CoA promotes lysine propionylation of the active nuclear form of SREBP2, thereby increasing its stability and transcriptional activity independently of canonical proteolytic activation and cholesterol-mediated feedback inhibition. Sustained SREBP2 activation increases cholesterol synthesis and storage, which in turn fuels steroidogenesis and maintains AR signalling. Activated AR further amplifies lipid synthesis and accumulation, reinforcing a feedforward loop that supports tumour cell survival under metabolic and therapeutic stress. Notably, we identify isoleucine and valine catabolism as the primary source of P-CoA in PCa cells, directly linking branched-chain amino acid (BCAA) metabolism to cholesterol-driven oncogenic signalling. Collectively, our findings reveal a previously unrecognized P-CoA–SREBP2–AR regulatory axis in PCa and suggest that targeting P-CoA production may represent a therapeutic strategy to disrupt cholesterol metabolism and impede disease progression.

## Results

### Elevation of propionyl-CoA and propionylcarnitine pools promotes prostate tumour growth and colonization

**Extended Data Fig. 1:**
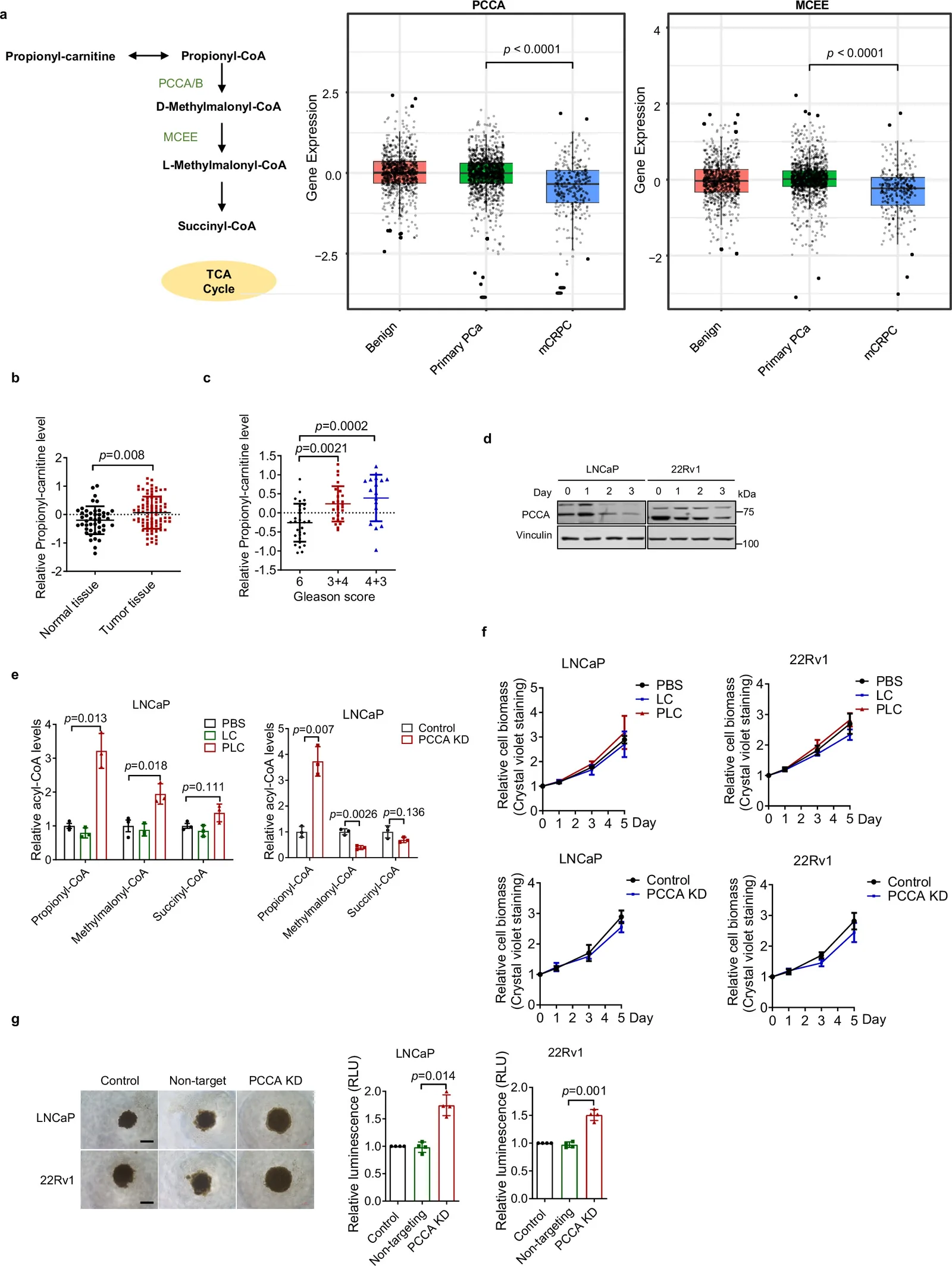
Propionate metabolism pathway activity during prostate cancer progression and effects of P-CoA elevation on prostate tumour cell proliferation and colony formation. **a**, Schematic representation of the propionate metabolism pathway and its associated genes. Gene expression levels were compared across benign prostate, primary prostate cancer (Primary PCa), and metastatic castration-resistant prostate cancer (mCRPC) samples. The centre line indicates the median; box bounds indicate the 25th and 75th percentiles; whiskers extend to the most extreme values within 1.5× the interquartile range (benign, n = 673; primary PCa, *n* = 1061; mCRPC, *n* = 260). **b**, Tumour tissue metabolomics data were reanalysed to compare propionylcarnitine (PLC) levels between normal prostate (*n* = 46) and tumour tissues (*n* = 91). **c**, Correlation between PLC levels and Gleason scores in prostate tumour tissues was examined (Gleason 6, *n* = 29; Gleason 3 + 4, *n* = 25; Gleason 4 + 3, *n* = 18). **d**, Tumour cells were transfected with siRNA targeting PCCA, and PCCA protein levels were measured at various time points post-transfection to assess knockdown efficiency. The western blots shown are representative of three independent experiments with similar results. **e**, Tumour cells were treated with LC or PLC for 2 or 3 days following PCCA knockdown, and intracellular acyl-CoA levels were quantified (*n* = 3). **f**, Tumour cells were treated with LC or PLC, or transfected with siRNA targeting PCCA. Cell biomass was measured by crystal violet staining over a 5-day period to assess proliferation (*n* = 3). **g**, Tumour cells with PCCA knockdown were cultured as spheroids, and spheroid biomass was quantified by CellTiter-Glo (*n* = 4). Data are presented as mea*n* ± s.d. from independent replicates. Statistical significance was assessed using two-tailed unpaired t-test (**a, b, e**); two-tailed paired t-test (**g**); one-way ANOVA (**c**) and two-way ANOVA (**f**). Scale bars: 100 μm (**g**). Source data

To explore the role of P-CoA metabolism in PCa progression, we began by analysing the expression of key enzymes involved in the propionate catabolic pathway across primary and metastatic prostate tumours^19^. Expression of propionyl-CoA carboxylase subunit α (PCCA) and methylmalonyl-CoA epimerase, two critical enzymes responsible for mitochondrial P-CoA catabolism, was significantly downregulated in metastatic CRPC (mCRPC) compared with hormone-sensitive primary tumours (Extended Data Fig. 1a). This downregulation theoretically impairs the conversion of P-CoA into succinyl-CoA, thereby reducing its entry into the tricarboxylic acid (TCA) cycle. In support of this hypothesis, levels of propionyl-L-carnitine (PLC), which interconverts with P-CoA and reflects its intracellular abundance, were significantly higher in tumour tissues than in healthy prostate, with a positive correlation to Gleason score, a clinical indicator of tumour aggressiveness and metastatic potential (Extended Data Fig. 1b,c)^20^. These findings suggest that prostate tumours may suppress P-CoA catabolism as a metabolic adaptation during progression, allowing P-CoA to engage in alternative pro-tumorigenic functions.

**Fig. 1:**
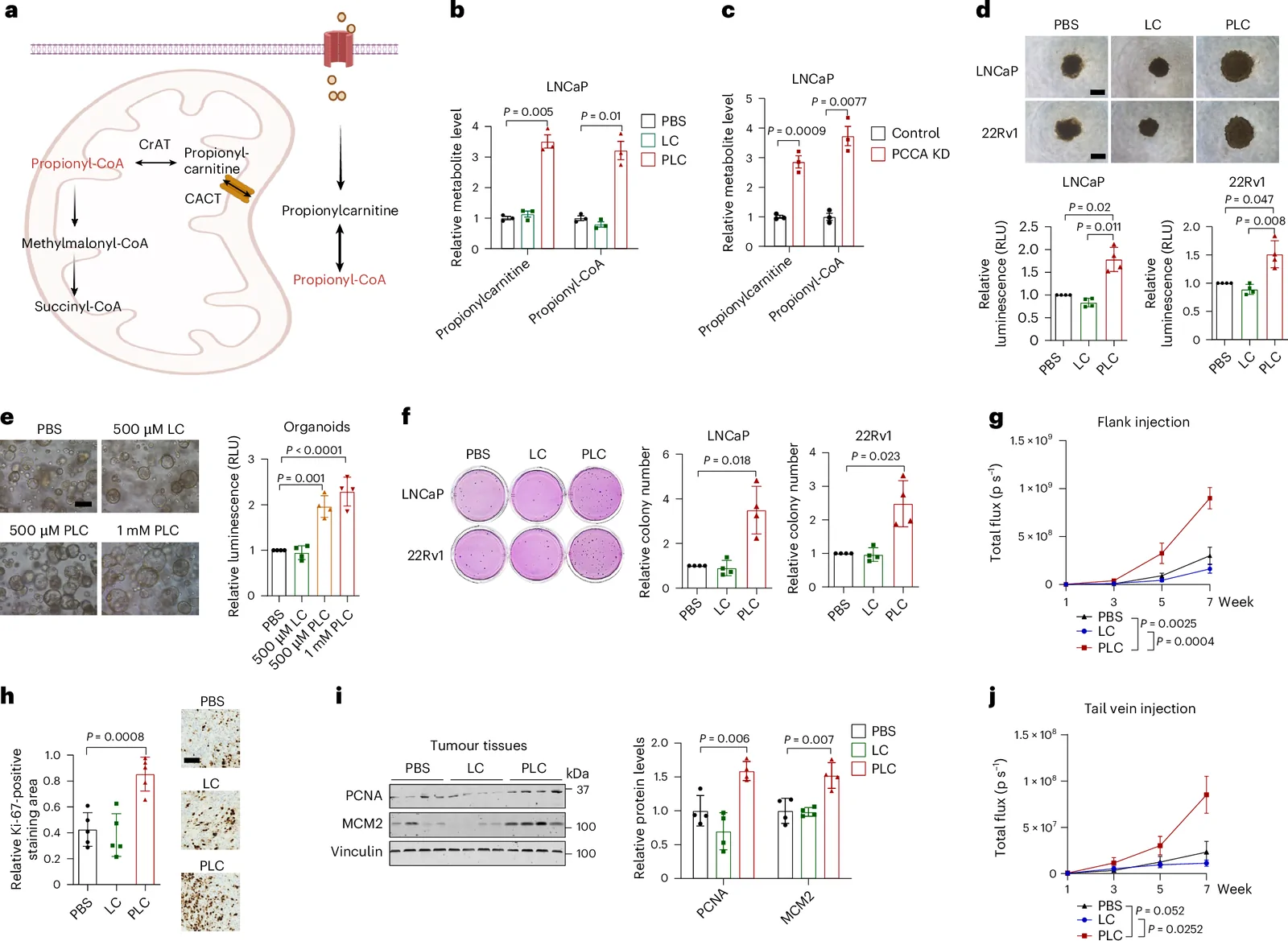
Elevated propionyl-CoA promotes prostate cancer cell growth and lung colonization. **a**, Schematic illustrating the reversible conversion between PLC and P-CoA and their dynamic equilibrium. **b**, LNCaP cells were treated with PBS, LC or PLC for 2 days, and intracellular levels of PLC and P-CoA were measured by LC–MS (*n* = 3). **c**, *PCCA* was knocked down in LNCaP cells using siRNA for 3 days, followed by measurement of intracellular PLC and P-CoA levels (*n* = 3). **d**, LNCaP cells were pretreated with LC or PLC for 5 days and then seeded to form spheroids without further treatment. Spheroid biomass was quantified using CellTiter-Glo (*n* = 4). **e**, Mouse prostate organoids were treated with LC or PLC for 9 days. Representative images are shown, and organoid biomass was quantified using CellTiter-Glo (*n* = 4). **f**, PCa cells were pretreated with the indicated metabolites for 5 days and then embedded in soft agar without further treatment to assess colony formation. Representative images and colony quantification are shown (*n* = 4). **g**–**i**, LNCaP cells were treated with LC or PLC for 7 days and then injected subcutaneously into nude mice. Tumour growth was monitored (**g**; *n* = 6–7 mice per group). Excised tumours were analysed for Ki-67 expression by immunohistochemistry (**h**; *n* = 5 tumours from 5 mice per group) and for the indicated markers of tumour aggressiveness by western blotting of total tumour lysates (**i**; *n* = 4 tumours from 4 mice per group). **j**, LNCaP cells pretreated with LC or PLC for 7 days were injected into nude mice through the tail vein, and lung colonization was assessed (*n* = 6–7 mice per group). Unless otherwise indicated, LC and PLC were used at 200 μM. Data are presented as mean ± s.d. from independent experiments or animals, as indicated. Statistical significance was assessed by two-sided unpaired *t*-test (**b**,**c**); one-way ANOVA (**d**–**f**,**h**,**i**); and two-way ANOVA (**g**,**j**). Scale bars, 100 μm (**d**,**h**), 300 μm (**e**). See also Extended Data Fig. 1. Source data

To investigate the functional consequences of P-CoA accumulation, we employed both pharmacological and genetic approaches to elevate intracellular P-CoA. We first supplemented cells with PLC, a stable and water-soluble molecule that enters cells via carnitine transporters and is rapidly converted to P-CoA (Fig. 1a). PLC treatment increased intracellular levels of both PLC and P-CoA to a similar extent (Fig. 1b). Because the concentrations required to elevate P-CoA exceeded typical serum levels (0.5–5 µM)^21^, we complemented this approach by knocking down *PCCA*, which blocks P-CoA conversion to methylmalonyl-CoA. This genetic perturbation also led to a comparable increase in intracellular P-CoA and PLC levels (Fig. 1c and Extended Data Fig. 1d), consistent with coordinated changes in the intracellular PLC and P-CoA pools. Due to the stability and ease of quantification of PLC, we used PLC levels as a surrogate marker for the P-CoA/PLC pool in subsequent experiments. Notably, while both approaches elevated P-CoA, they had distinct effects on downstream metabolites such as methylmalonyl-CoA and succinyl-CoA (Extended Data Fig. 1e), which are known to influence protein post-translational modifications^12^. These differences allowed us to distinguish P-CoA-specific effects from those potentially mediated by downstream catabolites.

To control for the potential impact of carnitine itself, L-carnitine (LC) was used as a control. Neither PLC supplementation nor *PCCA* knockdown significantly altered proliferation of LNCaP or 22Rv1 cells in standard two-dimensional (2D) monolayer culture (Extended Data Fig. 1f). However, in three-dimensional (3D) spheroid and organoid models, which better mimic the metabolic stressors of the tumour microenvironment, such as hypoxia and nutrient limitation^22^, both PLC treatment and *PCCA* knockdown markedly enhanced spheroid growth and colony formation (Fig. 1d–f and Extended Data Fig. 1g). These results suggest that elevated P-CoA confers a selective advantage under metabolically stressful conditions.

To assess the in vivo relevance of this pathway and determine whether elevated P-CoA levels can drive tumour progression, we used LNCaP cells, which are androgen-dependent. Cells were pretreated with PLC and implanted into nude mice. PLC pretreatment of the implanted cells significantly promoted tumour growth compared with controls (Fig. 1g). Tumours derived from PLC-treated cells displayed elevated expression of proliferation markers including Ki-67, proliferating cell nuclear antigen (PCNA) and minichromosome maintenance protein 2 (MCM2), indicative of increased mitotic activity and tumour aggressiveness (Fig. 1h,i). Furthermore, PLC-treated cells exhibited enhanced colonization of lung tissue following tail vein injection, demonstrating a heightened metastatic capacity (Fig. 1j). Collectively, these findings demonstrate that elevation of the P-CoA/PLC pool promotes prostate tumour growth and colonization.

### Isoleucine and valine catabolism is the primary source of propionyl-CoA in prostate cancer cells

**Fig. 2:**
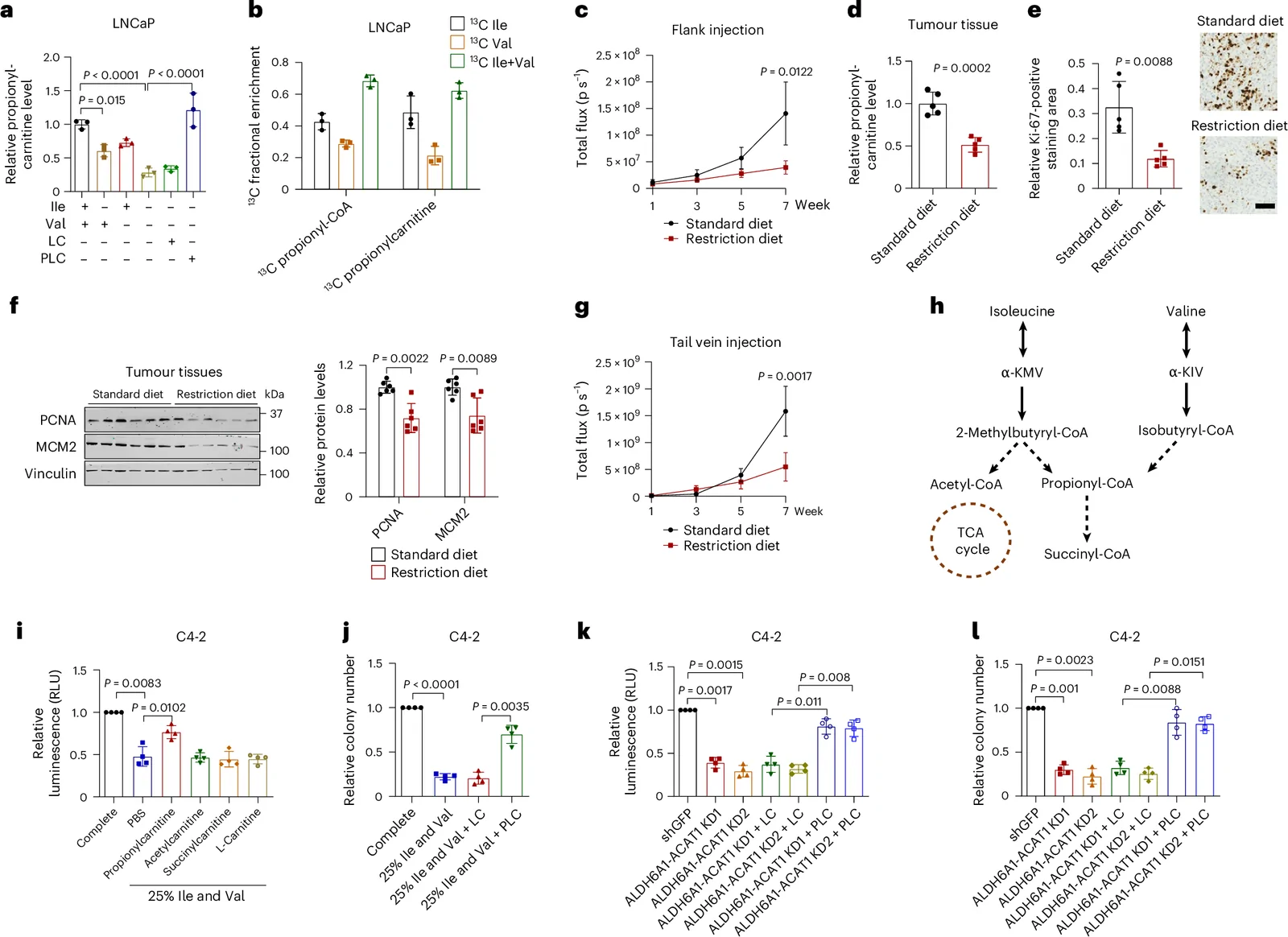
Isoleucine and valine catabolism is a major source of P-CoA in prostate cancer cells. **a**, Isoleucine, valine, or both amino acids were removed from the cell culture medium. Cells were supplemented with LC or PLC for 3 days, and intracellular PLC levels were measured (*n* = 3). **b**, LNCaP cells were incubated with medium containing ^13^C-labelled isoleucine and valine for 24 h. Incorporation into P-CoA and PLC was measured by LC–MS, and the fractions of ^13^C-labelled to total P-CoA and PLC were calculated (*n* = 3). **c**–**f**, C4-2 cells were implanted subcutaneously into nude mice, which were then fed either a complete control diet or an isoleucine- and valine-restricted diet for 7 weeks. Tumour growth was monitored during the dietary intervention (**c**; *n* = 8–9 mice per group). Resected tumours were analysed for PLC levels (**d**; *n* = 5 tumours from 5 mice per group), Ki-67 expression by immunohistochemistry (**e**; *n* = 5 tumours from 5 mice per group), and the indicated markers of tumour aggressiveness by western blotting of total tumour lysates (**f**; *n* = 6 tumours from 6 mice per group). **g**, C4-2 cells were injected into nude mice through the tail vein. Mice were fed either the complete control diet or the isoleucine- and valine-restricted diet for 7 weeks, and lung colonization was assessed (*n* = 8–9 mice per group). **h**, Schematic illustrating isoleucine and valine catabolism and the entry of their metabolic products into the TCA cycle through acetyl-CoA and succinyl-CoA. **i**–**l**, Tumour cells were cultured in medium containing 25% of the standard isoleucine and valine concentrations or were subjected to *ALDH6A1* or *ACAT1* knockdown. Cells were supplemented with the indicated acylcarnitines or control metabolites for 3 days and then cultured under spheroid-forming conditions (**i**,**k**; *n* = 4) or embedded in soft agar to assess colony formation (**j**,**l**; *n* = 4). All acylcarnitines and LC were used at 200 μM unless otherwise noted. Data are shown as mean ± s.d. from independent biological replicates. For the animal studies in **c** and **g**, *n* denotes the number of mice per group, with each mouse representing an independent biological replicate. Statistical significance was assessed by two-tailed unpaired *t*-test (**d**–**f**); two-way ANOVA (**c**,**g**); and one-way ANOVA (**a**,**i**–**l**). Scale bar, 100 μm (**e**). See also Extended Data Fig. 2. Source data

**Extended Data Fig. 2:**
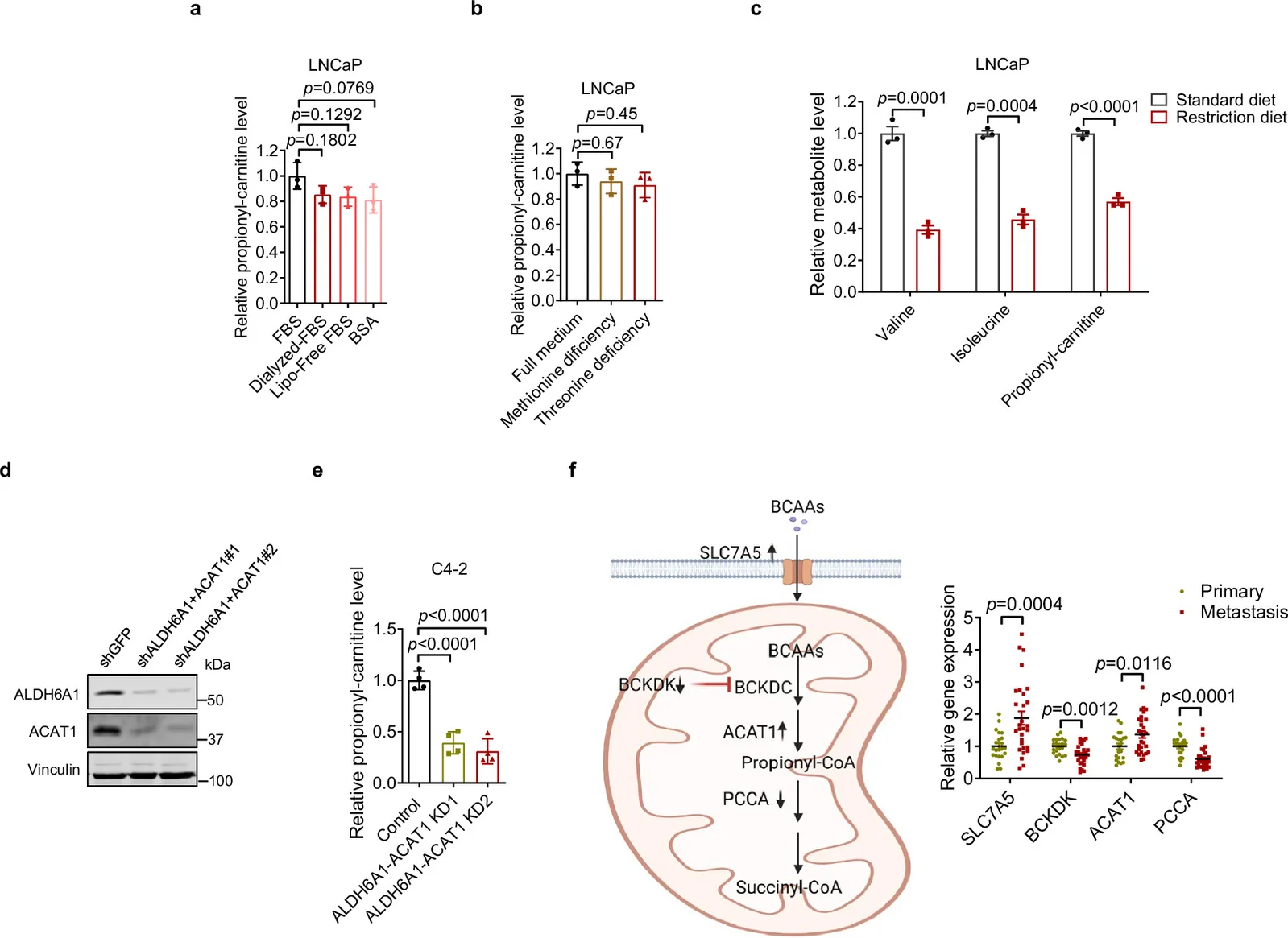
Isoleucine and valine are major nutrient sources for P-CoA production. **a**, LNCaP cells were cultured for 2 days in medium supplemented with 10% complete FBS, 10% dialysed FBS, 10% lipoprotein-depleted FBS, or 1% BSA, and cellular PLC levels were measured (*n* = 3). **b**, Methionine or threonine was individually removed from the culture medium, and PLC levels were measured after 3 days (*n* = 3). **c**, Nude male mice were fed an isoleucine- and valine-restricted diet for 1 week, as described in the Methods, and serum metabolite levels were measured by targeted metabolomics (*n* = 3 mice). **d, e**, ACAT1 and ALDH6A1 were simultaneously knocked down in C4-2 cells. Protein levels were assessed to confirm knockdown efficiency (**d**). The western blots shown in d are representative of three independent experiments with similar results; cellular PLC levels were quantified after 5 days (**e**; *n* = 4). **f**, Previously published gene expression data from prostate cancer tissues were reanalysed to compare the expression of enzymes involved in P-CoA synthesis and catabolism between primary localized prostate cancer (*n* = 22) and castration-resistant bone metastatic prostate cancer (*n* = 29). Data are presented as mea*n* ± s.d. from independent replicates. Statistical significance was assessed using one-way ANOVA (**a, b, e**); two-tailed unpaired t-tests (**c, f**). Source data

P-CoA can be generated from multiple metabolic inputs, including the breakdown of odd-chain fatty acids, cholesterol side chains, propionate and certain amino acids such as isoleucine, valine, threonine and methionine^16^. To identify the predominant source of P-CoA in PCa cells, we systematically manipulated each potential contributor and measured intracellular PLC levels in LNCaP cells. Replacing fetal bovine serum (FBS) with dialysed FBS, lipid-depleted FBS or lipid-free bovine serum albumin (BSA) caused only a minor reduction in PLC levels (Extended Data Fig. 2a), suggesting that exogenous serum lipids are not the major source of P-CoA in vitro. We next focused on amino acid catabolism. Among the known P-CoA-producing amino acids, removal of isoleucine or valine from the culture medium significantly reduced intracellular PLC levels, whereas depletion of threonine or methionine had a minimal effect (Fig. 2a and Extended Data Fig. 2b). To validate these findings, we conducted ^13^C-isotope tracing with labelled isoleucine and valine. Together, these two amino acids accounted for approximately 70% of the total P-CoA and PLC pool (Fig. 2b), identifying them as the principal contributors to P-CoA production in PCa cells.

To assess this metabolic dependency in vivo, we designed a dietary intervention that restricted isoleucine and valine to 25% of their standard levels, while compensating with other amino acids to maintain total protein-derived caloric intake. This restriction diet significantly decreased serum isoleucine, valine and PLC levels (Extended Data Fig. 2c). To assess the impact of this diet on tumour progression, we used C4-2 cells, which are derived from castration-resistant LNCaP xenografts and exhibit enhanced tumour growth and colonization compared with parental LNCaP cells, thereby providing a more robust model to observe differences induced by isoleucine and valine restriction. In xenograft models, this restriction diet markedly suppressed tumour growth (Fig. 2c). Tumours from restricted mice exhibited decreased PLC levels and reduced expression of proliferation markers Ki-67, PCNA and MCM2 (Fig. 2d–f), along with impaired lung colonization potential (Fig. 2g).

Because isoleucine and valine catabolism ultimately feeds into the TCA cycle via acetyl-CoA and succinyl-CoA (Fig. 2h), we next asked whether the tumour-suppressive effects observed under BCAA restriction were specifically due to reduced P-CoA levels or instead reflected a general reduction in TCA cycle intermediates. Notably, among the tested acylcarnitines, only PLC was able to restore spheroid growth under amino acid restriction (Fig. 2i), indicating a specific requirement for P-CoA. Consistently, PLC supplementation also partially rescued colony formation under isoleucine and valine deprivation (Fig. 2j).

To rule out potential confounding effects from reduced isoleucine and valine availability for protein synthesis, we knocked down *ACAT1* and *ALDH6A1*, which catalyse terminal steps in isoleucine and valine catabolism, respectively, leading directly to P-CoA production. Knocking down these two enzymes led to reduced PLC levels (Extended Data Fig. 2d,e) and inhibited spheroid and colony formation, which could be partially rescued by PLC (Fig. 2k,l). Together, these data support a model in which isoleucine and valine drive PCa progression, at least in part through P-CoA production.

Finally, transcriptomic analysis of patient samples revealed a gene expression signature consistent with enhanced BCAA catabolism and suppressed P-CoA degradation in advanced disease. Expression of *SLC7A5*, a BCAA transporter, and *ACAT1* was elevated in metastatic tumours, while expression of *BCKDK*, a negative regulator of BCAA catabolism, and *PCCA* was reduced (Extended Data Fig. 2f). These alterations support a model in which BCAA-driven P-CoA production is upregulated during PCa progression. Consequently, restricting isoleucine and valine availability may inhibit this adaptive elevation of P-CoA, thereby suppressing tumour growth and metastatic potential.

### Propionyl-CoA elevation primarily stimulates cholesterol synthesis

**Fig. 3:**
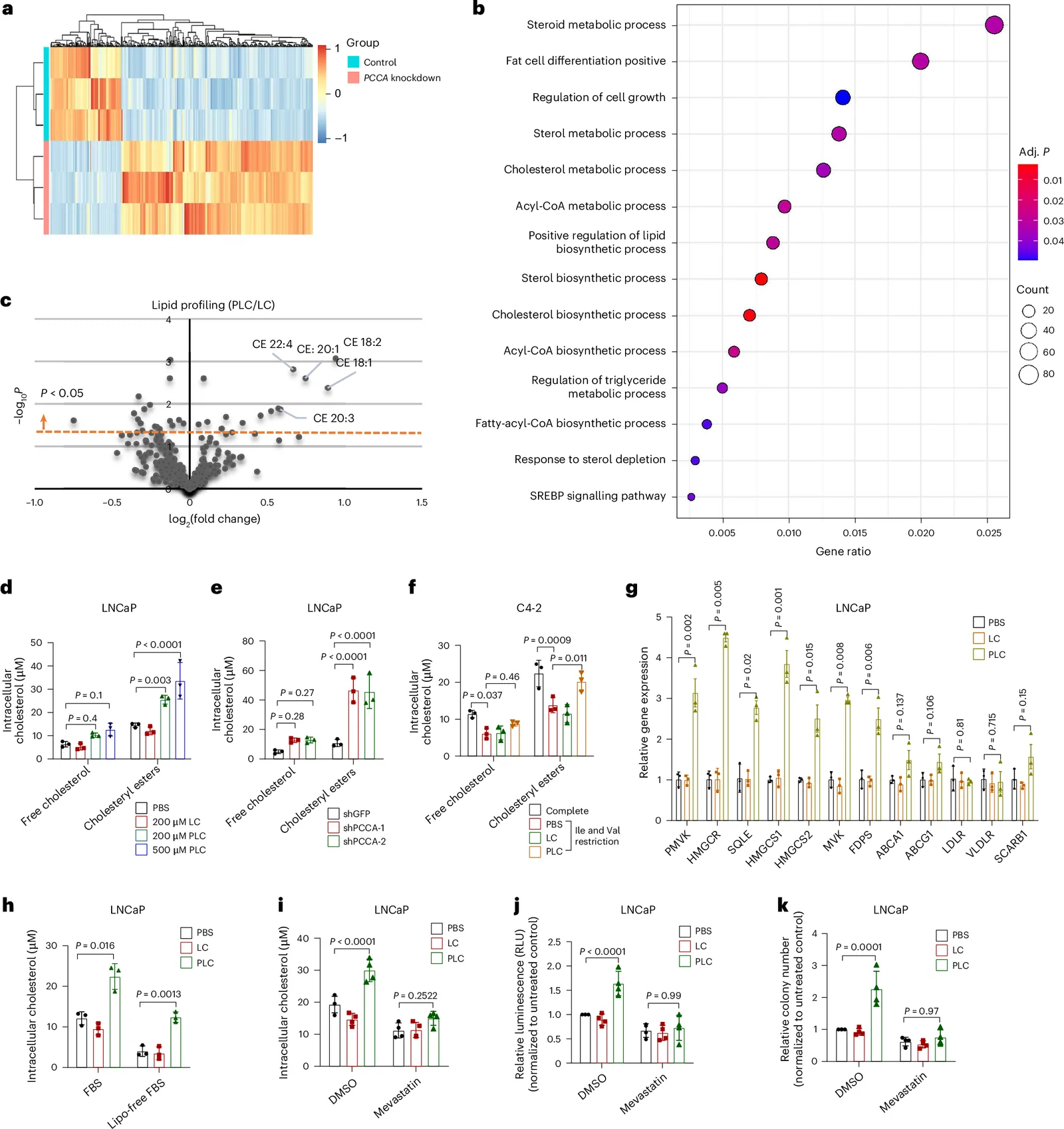
P-CoA promotes cholesterol synthesis and esterification. **a**,**b**, RNA sequencing was performed on LNCaP cells 2 days after *PCCA* knockdown (*n* = 3). A heatmap shows the 402 most variably expressed genes following *PCCA* knockdown (**a**). Pathway enrichment analysis was performed using these 402 genes (**b**). **c**, LNCaP cells were treated with LC or PLC for 3 days, followed by lipidomic profiling to assess global lipid changes (*n* = 3). **d**,**e**, Free cholesterol and cholesteryl ester levels were quantified in LNCaP cells treated with LC or PLC for 3 days (**d**; *n* = 3) or following *PCCA* knockdown for 3 days (**e**; *n* = 3). **f**, LNCaP cells were cultured in isoleucine- and valine-deficient medium supplemented with LC or PLC. Free cholesterol and cholesteryl ester levels were measured after 3 days (*n* = 3). **g**, mRNA levels of genes involved in cholesterol synthesis and transport were measured in LNCaP cells treated with LC or PLC for 2 days (*n* = 3). **h**, LNCaP cells were cultured in medium containing either 10% complete FBS or 10% lipoprotein-deficient FBS and treated with LC or PLC for 3 days. Total cholesterol levels were measured (*n* = 3). **i**, LNCaP cells were treated with LC or PLC in the presence or absence of mevastatin for 3 days, and total cholesterol levels were quantified (*n* = 4). **j**,**k**, Cells were pretreated with LC or PLC, with or without mevastatin, for 5 days and then cultured under spheroid-forming conditions (**j**; *n* = 4) or embedded in soft agar to assess colony formation (**k**; *n* = 4). All treatments used LC and PLC at 200 μM unless otherwise specified. Data are presented as mean ± s.d. from independent replicates. For pathway enrichment analysis in **b**, statistical significance was assessed using a one-sided hypergeometric test, with *P* values adjusted for multiple comparisons using the Benjamini–Hochberg method. Statistical significance in the remaining panels was assessed using two-sided unpaired *t*-tests (**c**,**g**), one-way ANOVA (**d**–**f**) or two-way ANOVA (**h**–**k**). See also Extended Data Fig. 3 and Supplementary Table 1. Adj., adjusted. Source data

**Extended Data Fig. 3:**
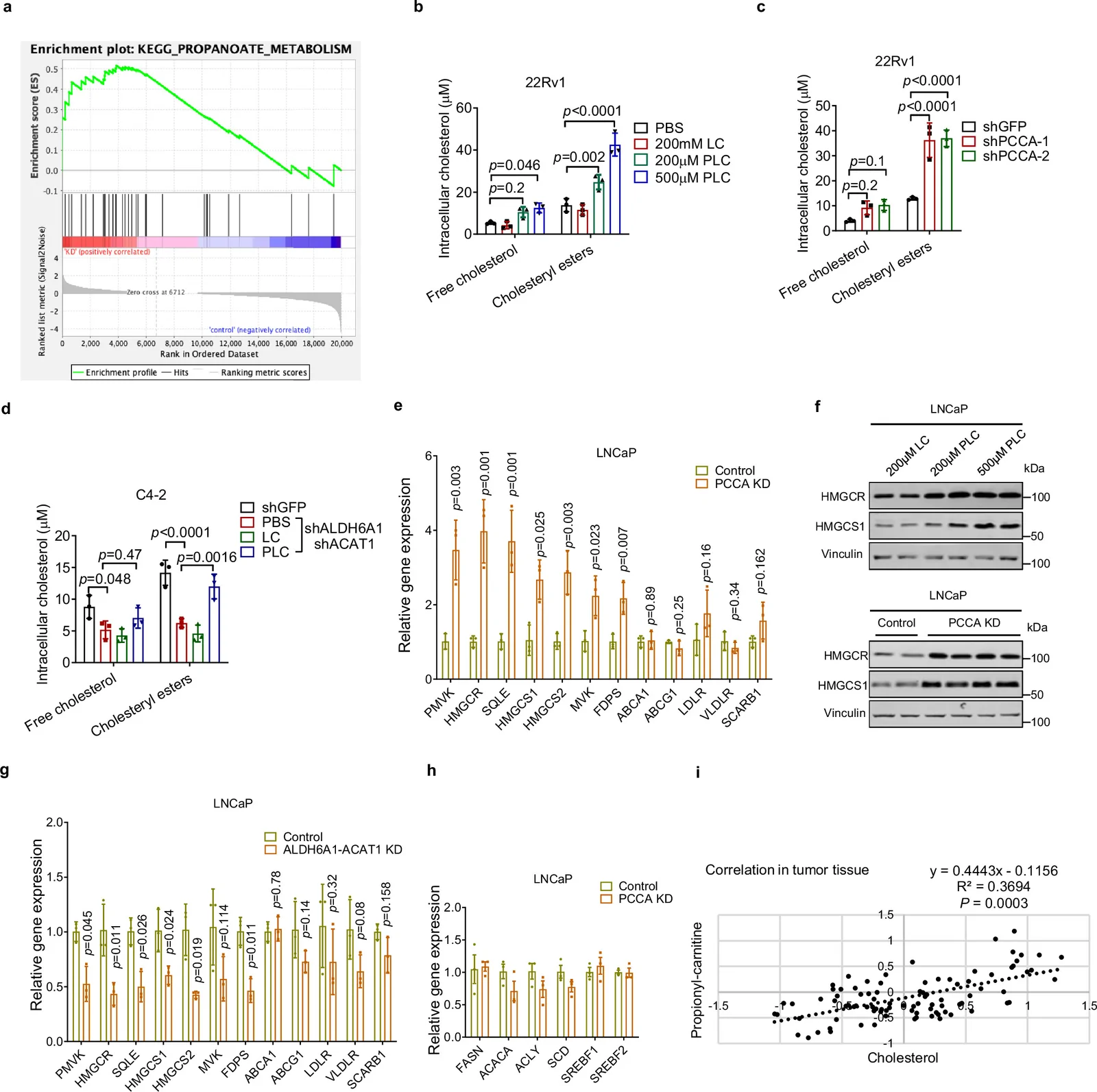
P-CoA regulates cholesterol synthesis. **a**, Pathway enrichment analysis identified alterations in the propionate metabolism pathway in LNCaP cells following PCCA knockdown. **b, c**, Free cholesterol and cholesteryl ester levels were quantified in 22Rv1 cells treated with LC or PLC for 3 days (**b**; *n* = 3), or following PCCA knockdown for 3 days (**c**; *n* = 3). **d**, ALDH6A1 and ACAT1 were knocked down in C4-2 cells, followed by LC or PLC treatment for 3 days. Cholesterol and ester levels were quantified (*n* = 3). **e**, mRNA levels of genes involved in cholesterol synthesis and transport were measured in LNCaP cells 2 days after PCCA knockdown (*n* = 3). **f**, Protein levels of enzymes involved in cholesterol synthesis and esterification were assessed by western blot in tumour cells transfected with siRNA or treated with LC or PLC for 3 days. The western blots shown are representative of three independent experiments with similar results. **g**, ALDH6A1 and ACAT1 were knocked down in LNCaP cells for 4 days, and mRNA levels of cholesterol synthesis and transport genes were quantified (*n* = 3). **h**, LNCaP cells were transfected with siRNA targeting PCCA for 2 days, and mRNA levels of genes involved in fatty acid synthesis were measured (*n* = 3). **i**, The correlation between tissue propionylcarnitine and cholesterol levels was assessed in prostate tumour samples (*n* = 91 samples). Data are presented as mea*n* ± s.d. from independent replicates. Statistical significance was assessed using one-way ANOVA (**b-d**); two-tailed unpaired t-tests (**e, g, h**); two-sided Pearson’s correlation coefficient (**i**). Source data

To elucidate the mechanisms by which elevated P-CoA promotes PCa progression, we performed RNA sequencing in LNCaP cells following *PCCA* knockdown to capture transcriptional responses driven by P-CoA accumulation. As expected, *PCCA* knockdown disrupted the propionate metabolism pathway (Fig. 3a and Extended Data Fig. 3a), leading to broad transcriptional reprogramming. Pathway enrichment analysis revealed a striking upregulation of genes involved in cholesterol biosynthesis, steroidogenesis and lipid metabolism (Fig. 3b), suggesting a central role for P-CoA in rewiring lipid regulatory programmes.

To determine whether these transcriptional changes translated into metabolic alterations, we conducted lipidomic profiling of LNCaP cells treated with PLC or LC. Metabolic changes became apparent after 3 days of PLC treatment, with cholesteryl esters representing the most significantly elevated lipid class (Fig. 3c and Supplementary Table 1). Both free and esterified cholesterol levels were increased by PLC, with a more pronounced rise in the esterified form (Fig. 3d and Extended Data Fig. 3b). A similar elevation in cholesterol levels was observed in *PCCA*-knockdown cells (Fig. 3e and Extended Data Fig. 3c). In contrast, suppression of P-CoA production, via isoleucine and valine restriction or knockdown of *ALDH6A1* and *ACAT1*, significantly reduced cholesterol levels. Notably, these reductions were partially reversed by PLC supplementation (Fig. 3f and Extended Data Fig. 3d), reinforcing the idea that P-CoA is required to sustain cholesterol biosynthesis in PCa cells.

To investigate whether the elevated cholesterol resulted from enhanced de novo synthesis or altered transport, we measured the expression of key genes involved in these processes. Both PLC treatment and *PCCA* knockdown robustly upregulated mRNA levels of cholesterol biosynthetic enzymes (Fig. 3g and Extended Data Fig. 3e), and this was corroborated at the protein level, particularly for HMGCR, the rate-limiting enzyme of cholesterol synthesis (Extended Data Fig. 3f). Conversely, *ALDH6A1* or *ACAT1* knockdown resulted in a marked downregulation of these same genes (Extended Data Fig. 3g), confirming that P-CoA generation is essential for maintaining the cholesterol synthesis programme in PCa cells. Notably, expression of cholesterol uptake and efflux transporters was largely unchanged in response to P-CoA manipulation (Fig. 3g and Extended Data Fig. 3e,g), indicating that the cholesterol increase was not due to altered expression of the transporters for import or export. Moreover, even under lipid-depleted conditions, tumour cells maintained high cholesterol levels when P-CoA was elevated (Fig. 3h), further supporting the conclusion that the cholesterol accumulation was primarily due to de novo synthesis.

To determine whether this cholesterol synthesis is functionally required for P-CoA-driven tumour phenotypes, we treated cells with mevastatin, an HMGCR inhibitor, to inhibit cholesterol synthesis. Mevastatin blocked the PLC-induced increase in cholesterol levels and significantly impaired spheroid growth and colony formation (Fig. 3i–k), demonstrating that de novo cholesterol synthesis is a critical mediator of the growth-promoting effects of P-CoA. Notably, while cholesterol biosynthesis genes were consistently upregulated by P-CoA elevation, genes involved in fatty acid synthesis were not significantly altered during early responses to *PCCA* knockdown (Extended Data Fig. 3h). This suggests a selective and specific link between P-CoA and the cholesterol biosynthetic programme.

Finally, to evaluate the clinical relevance of this mechanism, we analysed metabolomics data from human prostate tumour tissues. A strong positive correlation was observed between tissue PLC levels and total cholesterol content (Extended Data Fig. 3i), supporting an association between elevated P-CoA/PLC pools and cholesterol accumulation in human prostate tumours. Together, these findings identify cholesterol biosynthesis as a primary and selective downstream target of P-CoA elevation in PCa cells.

### Propionyl-CoA regulates SREBP2 stability and activity through lysine propionylation

Given the robust upregulation of cholesterol biosynthetic genes in response to elevated P-CoA, we hypothesized that this regulation occurs at the transcriptional level. Sterol regulatory element-binding proteins (SREBPs) are master transcription factors that control lipid metabolism, with SREBP2 specifically regulating genes involved in cholesterol synthesis and uptake^9^. SREBPs are initially synthesized as inactive precursors (P-SREBP1/2) and become transcriptionally active upon proteolytic cleavage by S1P and S2P in the Golgi, releasing their N-terminal fragments (N-SREBP1/2) that translocate to the nucleus.

**Fig. 4:**
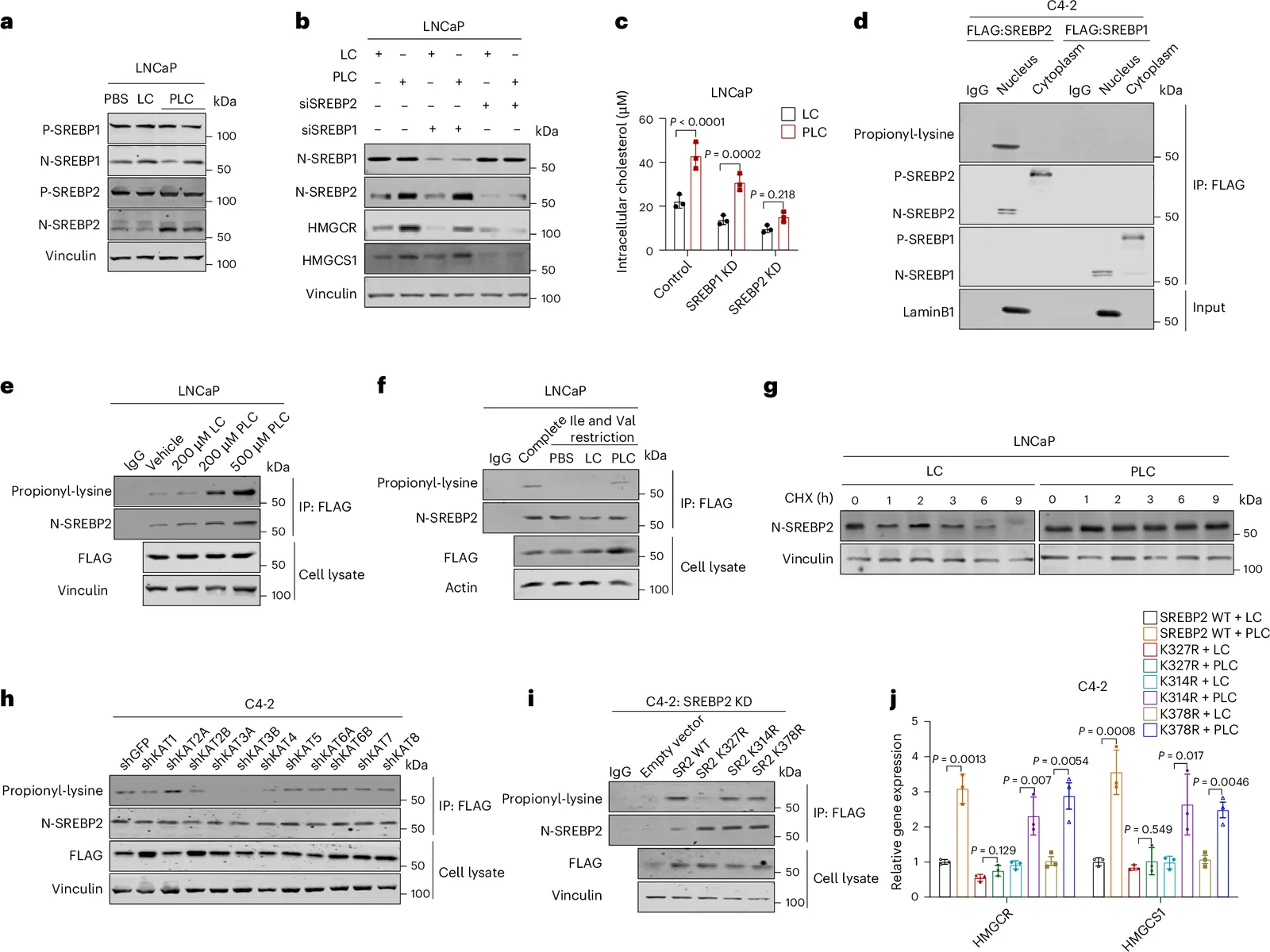
P-CoA regulates SREBP2 through lysine propionylation. **a**, LNCaP cells were treated with LC or PLC for 24 h, and protein levels of precursor (P−) and nuclear (N−) forms of SREBP1 and SREBP2 were assessed by western blotting. **b**,**c**, Tumour cells were transfected with siRNAs targeting *SREBF1* or *SREBF2* or a scramble control and treated with LC or PLC. After 3 days, protein levels of cleaved SREBPs and cholesterol biosynthetic enzymes were measured (**b**), and total cholesterol levels were quantified (**c**; *n* = 3). **d**, FLAG-tagged SREBP2 or SREBP1 was separately overexpressed in C4-2 cells, followed by PLC treatment for 24 hours. Nuclear and cytoplasmic fractions were prepared separately, and FLAG-tagged proteins were immunoprecipitated and analysed for lysine propionylation by western blotting. **e**, PCa cells overexpressing FLAG-tagged nuclear SREBP2 were treated with LC or PLC for 2 days. FLAG-tagged SREBP2 was immunoprecipitated and analysed for lysine propionylation. **f**, Tumour cells were cultured in isoleucine- and valine-deficient medium supplemented with LC or PLC for 2 days. FLAG-tagged SREBP2 was immunoprecipitated and assessed for lysine propionylation. **g**, PCa cells were treated with the indicated metabolites for 2 days, followed by a CHX chase assay to assess SREBP2 protein stability. **h**, Cells were infected with shRNAs targeting different histone acetyltransferases, followed by PLC treatment for 24 h. FLAG-tagged SREBP2 was immunoprecipitated and analysed for lysine propionylation. **i**,**j**, Endogenous SREBP2 was depleted using an shRNA targeting *SREBF2*, followed by re-expression of FLAG-tagged wild-type (WT) SREBP2 or the K314R, K327R, or K378R mutants. After treatment with PLC for 24 h, SREBP2 was immunoprecipitated and analysed for lysine propionylation (**i**). In LNCaP cells, the indicated SREBP2 constructs were re-expressed after endogenous SREBP2 depletion, followed by treatment with LC or PLC for 2 days. mRNA levels of cholesterol biosynthetic target genes were then measured by quantitative PCR (**j**; *n* = 3). Data are shown as mean ± s.d. from independent replicates. Statistical significance was assessed by two-way ANOVA (**c**,**j**). The western blots shown are representative of three independent experiments with similar results. See also Extended Data Figs. 4 and 5. Source data

**Extended Data Fig. 4:**
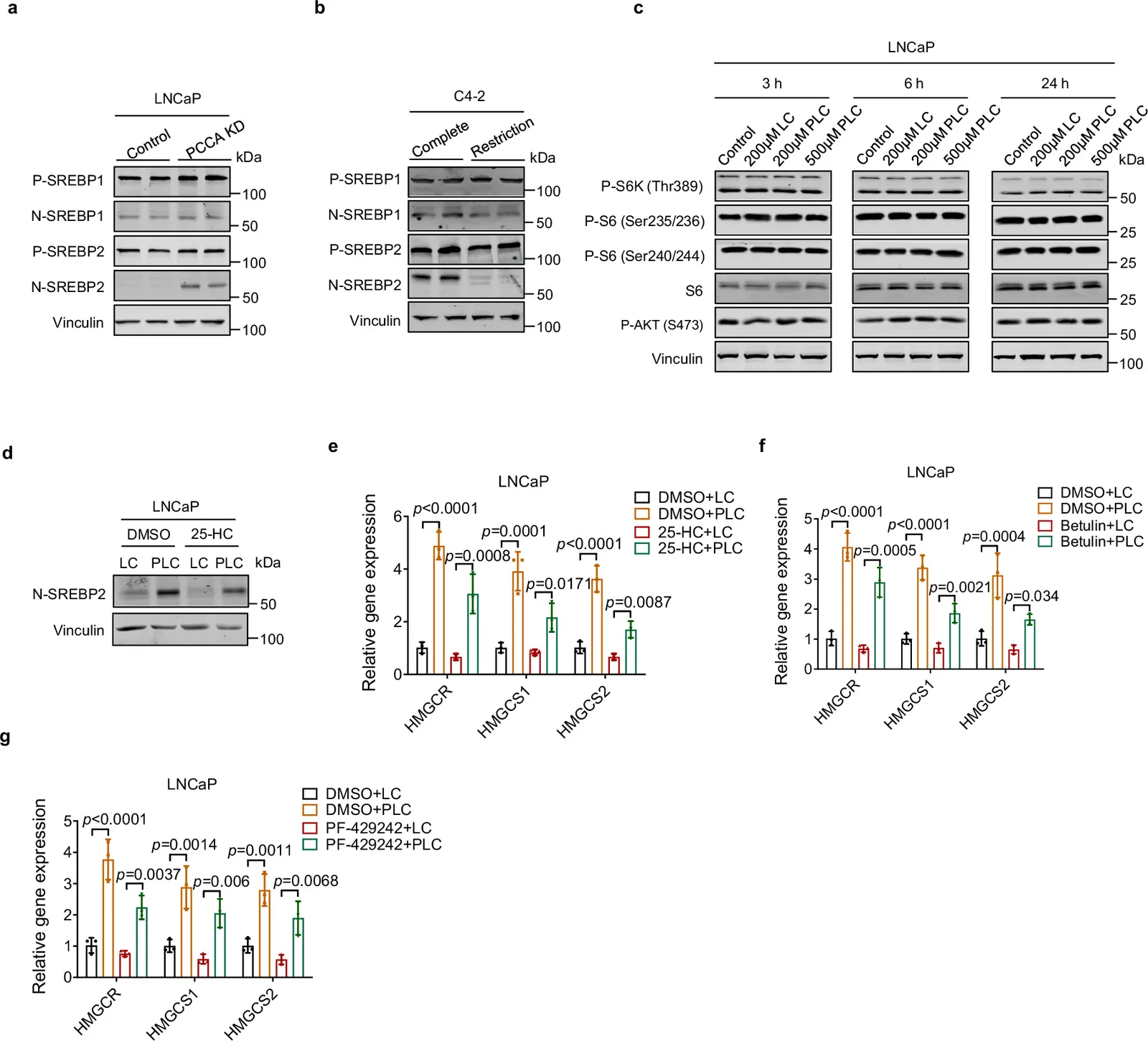
P-CoA regulates nuclear SREBP2 independently of mTOR signalling and the canonical translocation and cleavage pathways. **a**, LNCaP tumour cells were transfected with siRNA targeting PCCA for 2 days. Protein levels of both precursor (P−) and nuclear (N−) forms of SREBP1 and SREBP2 were examined by western blot. **b**, C4-2 cells were cultured in isoleucine- and valine-deficient medium for 3 days. Protein levels of both P- and N-SREBP1/2 were assessed. **c**, LNCaP tumour cells were treated with the indicated metabolites, and mTOR pathway activity was assessed at the indicated time points by western blotting for downstream pathway components. **d, e**, LNCaP cells were treated with 200 μM LC or PLC, with or without 5 μM 25-hydroxycholesterol (25-HC). Protein extracts were prepared after 24 h and analysed for cleaved SREBP2 (**d**), and mRNA levels of cholesterol synthesis-related enzymes were quantified after 2 days (**e**; *n* = 3). **f, g**, LNCaP cells were treated with 200 μM LC or PLC, with or without 2 μM Betulin (**f**) or 10 μM PF-429242 (**g**) for 2 days. mRNA levels of cholesterol synthesis-related genes were quantified (*n* = 3). Data are presented as mea*n* ± s.d. from independent replicates. Statistical significance in (**e-g**) was determined using two-way ANOVA. The western blots shown are representative of three independent experiments with similar results. Source data

We observed that both *PCCA* knockdown and PLC treatment selectively increased the nuclear, active form of N-SREBP2 without affecting P-SREBP2 or N-SREBP1 levels (Fig. 4a and Extended Data Fig. 4a). Conversely, restriction of isoleucine and valine decreased N-SREBP2 levels (Extended Data Fig. 4b), suggesting that SREBP2 activation responds specifically to intracellular P-CoA levels. To test whether SREBP2 is essential for P-CoA-driven cholesterol synthesis, we knocked down *SREBF2*, which abolished the P-CoA-induced upregulation of key cholesterol biosynthetic enzymes (Fig. 4b) as well as the accumulation of cholesterol (Fig. 4c). These data demonstrate that SREBP2 is required to mediate the cholesterol-promoting effects of P-CoA.

To elucidate how P-CoA increases N-SREBP2 levels, we first examined the mTOR signalling pathway, a known upstream regulator of SREBP activation^23^. However, PLC treatment did not alter mTOR activity at early time points when we observed the N-SREBP2 changes (Extended Data Fig. 4c), suggesting an mTOR-independent mechanism. Additionally, P-SREBP2 protein levels remained unchanged by P-CoA manipulation, ruling out transcriptional or translational regulation.

We next investigated whether P-CoA influences the cleavage activation of SREBP2. Usually, this process is tightly regulated by sterol-mediated feedback. For instance, 25-hydroxycholesterol (25-HC) binds insulin-induced gene and promotes its interaction with SREBP cleavage-activating protein (SCAP), thereby preventing ER-to-Golgi transport of the SCAP–SREBP2 complex^24^. However, 25-HC failed to block PLC-induced N-SREBP2 elevation or its target gene expression (Extended Data Fig. 4d,e). Similarly, betulin, another SCAP-binding inhibitor, did not inhibit SREBP2 activation by PLC (Extended Data Fig. 4f). Furthermore, inhibition of S1P protease also failed to suppress P-CoA-driven transcriptional activation of cholesterol synthesis genes (Extended Data Fig. 4g). Together, these findings indicate that P-CoA promotes SREBP2 activation via a non-canonical mechanism, independent of proteolytic cleavage or classical sterol-mediated feedback.

Given that nuclear SREBPs are subject to proteasomal degradation, which can be affected by post-translational modifications (PTMs), we hypothesized that P-CoA may stabilize N-SREBP2 via lysine propionylation, a PTM that uses P-CoA as a donor and is increasingly recognized as a functional modification^25^. To test this, we overexpressed SREBP1 or SREBP2 in LNCaP cells, treated cells with PLC, and assessed lysine propionylation by subcellular fractionation. Notably, lysine propionylation was detected exclusively on N-SREBP2, but not on N-SREBP1 or any precursor forms (Fig. 4d), providing a molecular basis for the selective effect of P-CoA on SREBP2.

**Extended Data Fig. 5:**
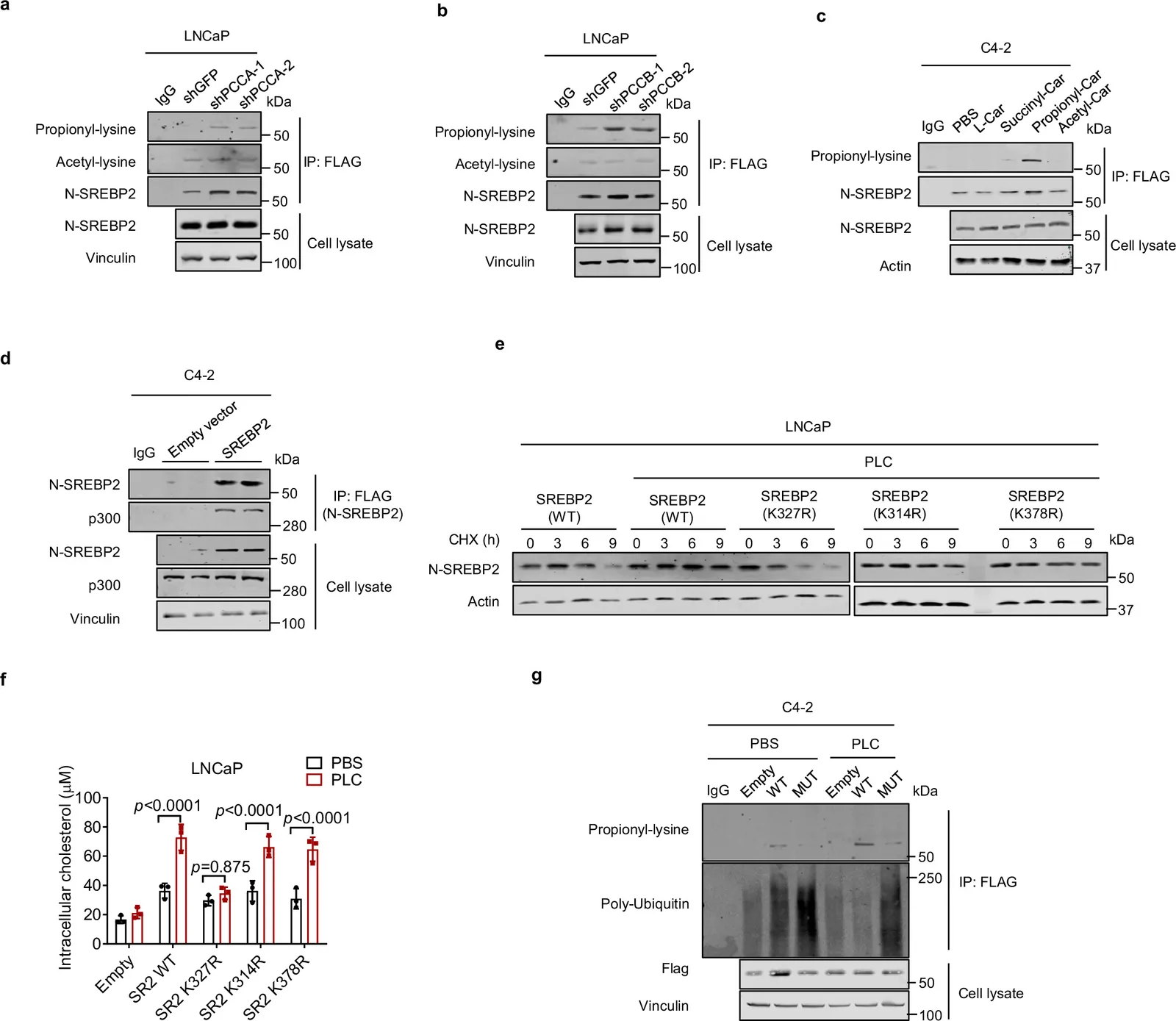
P-CoA regulates nuclear SREBP2 stability through lysine propionylation. **a,b**, FLAG-tagged SREBP2 was overexpressed in C4-2 cells, followed by knockdown of PCCA (**a**) or PCCB (**b**) for 2 days. SREBP2 was immunoprecipitated using anti-FLAG antibodies, and lysine propionylation levels were assessed by western blot. **c**, C4-2 cells overexpressing FLAG-tagged SREBP2 were treated with the indicated acylcarnitines for 2 days. FLAG-tagged SREBP2 was immunoprecipitated, and lysine propionylation was assessed by western blotting. **d**, C4-2 cells were transfected with FLAG-tagged SREBP2, and immunoprecipitation was performed using anti-FLAG affinity beads. Co-precipitated p300 was detected by western blot to assess protein–protein interaction. **e, f**, LNCaP cells were transfected with FLAG-tagged wild-type (WT), K327R, K314R, or K378R SREBP2 and treated with the indicated metabolites. SREBP2 protein stability was assessed by cycloheximide (CHX) chase assay after 2 days of treatment (**e**), and intracellular total cholesterol levels were measured after 3 days of treatment (**f**; *n* = 3). **g**, C4-2 cells were transfected with empty vector or FLAG-tagged WT or K327R mutant SREBP2. After 2 days of metabolite treatment, SREBP2 was immunoprecipitated and analysed for lysine propionylation and ubiquitylation by western blotting. Data are presented as mea*n* ± s.d. from independent replicates. Statistical significance in (**f**) was determined using two-way ANOVA. The western blots shown are representative of three independent experiments with similar results. Source data

P-CoA elevation, via PLC treatment or *PCCA/PCCB* knockdown, robustly increased N-SREBP2 propionylation (Fig. 4e and Extended Data Fig. 5a,b), without affecting its acetylation (Extended Data Fig. 5a,b), indicating specificity and independent regulation of the propionylation effect. Supplementation with other short-chain acylcarnitines failed to induce SREBP2 propionylation (Extended Data Fig. 5c), further confirming the specificity of this measurement. Moreover, culturing cells in isoleucine- and valine-deficient medium suppressed N-SREBP2 propionylation, which was restored by PLC supplementation (Fig. 4f), supporting the availability of BCAA-derived P-CoA for SREBP2 propionylation. Finally, cycloheximide chase assays demonstrated that PLC treatment stabilized N-SREBP2 protein levels (Fig. 4g), suggesting that lysine propionylation enhances N-SREBP2 stability.

To identify the lysine acetyltransferase responsible for this modification, we screened several candidates and found that depletion of KAT3A/CBP and KAT3B/p300 significantly reduced SREBP2 propionylation in PLC-treated C4-2 cells (Fig. 4h), implicating these enzymes as potential mediators of this modification. Supporting this, co-immunoprecipitation analysis detected an association between p300 and SREBP2 (Extended Data Fig. 5d), further reinforcing p300’s role in catalysing SREBP2 propionylation. To assess the functional relevance of SREBP2 propionylation in response to P-CoA elevation, we next sought to identify the key lysine residue(s) involved. We performed an in silico screening of the SREBP2 protein sequence (NP_004590.2), prioritizing lysines located within p300/CBP-preferred motifs (GK, KK, KR and KXK), with high solvent accessibility and evolutionary conservation^26,27^. K314, K327 and K378 emerged as top candidates. Among them, mutating K327 to arginine (K327R) significantly decreased PLC-induced SREBP2 propionylation (Fig. 4i). Moreover, the K327R mutation, but not mutations at the other tested lysine residues, abolished the stabilizing effect of PLC on SREBP2 (Extended Data Fig. 5e) and attenuated PLC-induced increases in cholesterol biosynthetic gene expression and intracellular cholesterol levels (Fig. 4j and Extended Data Fig. 5f). To explore the mechanism underlying this effect, we measured SREBP2 ubiquitylation alongside propionylation. Increased propionylation was generally associated with reduced ubiquitylation, whereas the K327R mutation blunted PLC-induced propionylation and was accompanied by elevated ubiquitylation of SREBP2 (Extended Data Fig. 5g), suggesting that K327-dependent SREBP2 propionylation promotes SREBP2 stability, at least in part, by antagonizing ubiquitylation. Taken together, these findings demonstrate that P-CoA enhances SREBP2 stability and transcriptional activity via site-specific lysine propionylation, providing a mechanistic link between altered metabolism and cholesterol biosynthesis in PCa cells.

### Propionyl-CoA-mediated cholesterol synthesis facilitates steroid production, sustains AR activity, and promotes androgen-deprivation resistance

**Fig. 5:**
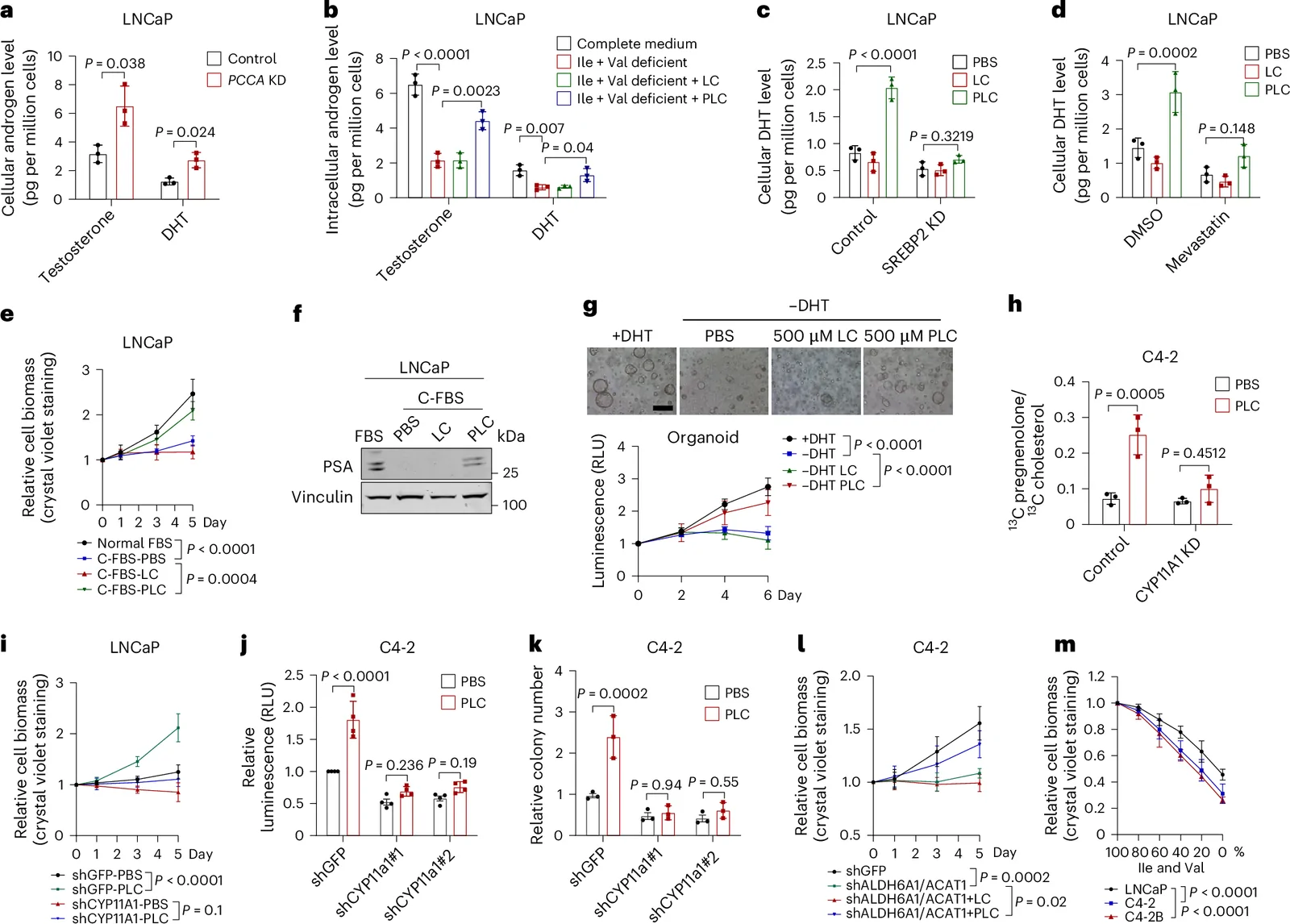
P-CoA promotes androgen synthesis and resistance to androgen deprivation. **a**, Intracellular testosterone and DHT levels in LNCaP cells 5 days after *PCCA* knockdown (*n* = 3). **b**, PCa cells were cultured in isoleucine- and valine-deficient medium supplemented with LC or PLC for 5 days, after which intracellular testosterone and DHT levels were quantified (*n* = 3). **c**,**d**, Tumour cells were transfected with *SREBF2* siRNA (**c**) or treated with mevastatin (**d**), followed by LC or PLC treatment for 5 days. DHT levels were then measured (*n* = 3). **e**,**f**, Tumour cells were pretreated with PBS, LC or PLC for 2 days and then transferred to androgen-deprived medium while maintaining the indicated treatments. Cell biomass was measured by crystal violet staining (**e**; *n* = 3), and PSA levels were assessed after 3 days (**f**). **g**, Prostate organoids were pretreated with LC or PLC for 4 days and then cultured under DHT-deprived conditions for an additional 6 days while the indicated treatments were maintained. Representative images are shown, and organoid biomass was measured over time (*n* = 3). **h**–**k**, *CYP11A1* was knocked down in LNCaP and C4-2 cells, followed by PLC treatment. Steroidogenesis was assessed using ^13^C-cholesterol tracing. After 2 days, intracellular ^13^C-cholesterol levels and the ratio of ^13^C-pregnenolone to ^13^C-cholesterol were measured (**h**; *n* = 3). Cells were subsequently transferred to androgen-deprived medium, and cell proliferation was monitored (**i**; *n* = 3). After 5 days, cells were cultured under spheroid-forming conditions (**j**; *n* = 4) or embedded in soft agar to assess colony formation (**k**; *n* = 3). **l**, *ALDH6A1* and *ACAT1* were knocked down in C4-2 cells, followed by LC or PLC treatment for 2 days. Cells were then transferred to androgen-deprived medium, and proliferation was assessed (*n* = 3). **m**, LNCaP, C4-2 and C4-2B cells were cultured for 5 days in medium containing progressively reduced concentrations of isoleucine and valine, and cell viability was measured (*n* = 3). Data are presented as mean ± s.d. from independent replicates. Western blots shown are representative of three independent experiments with similar results. Statistical significance was assessed using two-sided unpaired *t*-test (**a**), one-way ANOVA (**b**) and two-way ANOVA (**c**–**e**,**g**–**m**). Scale bar, 300 μm (**g**). See also Extended Data Fig. 6. Source data

**Extended Data Fig. 6:**
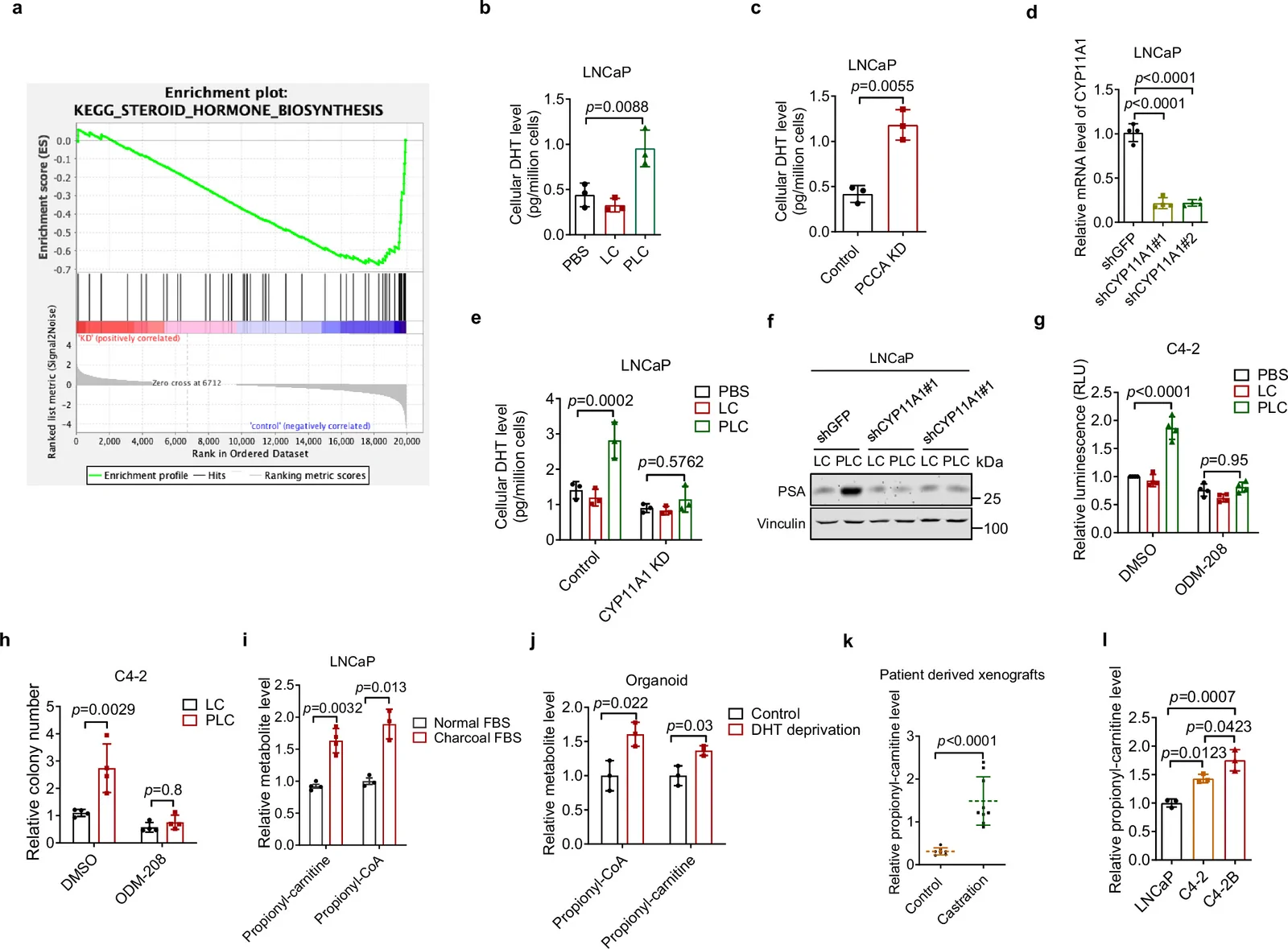
P-CoA promotes steroidogenesis by channelling cholesterol toward androgen synthesis under androgen-deprived conditions. **a**, Pathway enrichment analysis identified steroid biosynthesis as significantly enriched following PCCA knockdown in LNCaP cells. **b, c**, LNCaP cells were cultured in charcoal-stripped FBS, treated with LC or PLC, or transfected with siRNA targeting PCCA for 3 days. Intracellular DHT levels were measured (*n* = 3). **d–f**, CYP11A1 was knocked down in LNCaP cells. CYP11A1 mRNA levels were quantified after 2 days to confirm knockdown efficiency (**d**; *n* = 3). Cells were treated with LC or PLC for 5 days and intracellular DHT levels (**e**) and PSA protein levels were measured (**f**). The western blots shown are representative of three independent experiments with similar results. **g, h**, C4-2 cells were treated with LC or PLC, with or without ODM-208 for 5 days. Cells were then cultured as spheroids to assess biomass (**g**; *n* = 4) or in soft agar to assess colony formation (**h**; *n* = 4). **i**, LNCaP cells were cultured in charcoal-stripped FBS for 2 days. Intracellular P-CoA and PLC levels were measured (*n* = 3). **j**, Prostate organoids were cultured under either complete or DHT-deficient conditions for 3 days. PLC and P-CoA levels were quantified and normalized to cell number (*n* = 3). **k**, Previously published metabolomics data from CB17 SCID mouse xenografts with or without surgical castration were reanalysed to compare tumour tissue PLC levels (control, n = 7 tumours from 7 mice; castrated, *n* = 9 tumours from 9 mice). **l**, PLC levels were measured and normalized to cell number in LNCaP, C4-2, and C4-2B prostate cancer cell lines (*n* = 3). Data are presented as mea*n* ± s.d. from independent replicates. Statistical significance was determined by two-tailed unpaired t-test (**c, i, j, k**), one-way ANOVA (**b, d, l**), and two-way ANOVA (**e, g, h**). Source data

We next sought to understand the functional consequences of P-CoA-mediated cholesterol synthesis. Cholesterol serves as a precursor for multiple critical processes, including steroidogenesis, which is particularly important in steroid hormone-dependent cancers such as PCa. While steroid hormone production is typically restricted to specialized endocrine tissues, PCa has been shown to exhibit enhanced steroidogenic activity compared with normal prostate tissue, particularly in castration-resistant stages of the disease^28^. Consistent with this, our data showed that knockdown of *PCCA* activated the steroid hormone biosynthesis pathway (Extended Data Fig. 6a). In LNCaP cells, *PCCA* knockdown significantly increased intracellular levels of testosterone and dihydrotestosterone (DHT) (Fig. 5a). Conversely, isoleucine and valine restriction reduced these androgen levels, which could be rescued by supplementing PLC (Fig. 5b).

To determine whether this increase was due to enhanced de novo synthesis or uptake from the medium, we cultured cells in charcoal-stripped FBS, which lacks exogenous steroids. PLC treatment or *PCCA* knockdown still increased intracellular androgen levels under these conditions, indicating that the effect is driven by de novo synthesis (Extended Data Fig. 6b,c). These findings suggest that elevated P-CoA enhances endogenous androgen production, in parallel with elevation of cholesterol synthesis.

We next asked whether this increase in steroidogenesis was downstream of SREBP2-mediated cholesterol synthesis. *SREBF2* knockdown abolished the PLC-induced increase in intracellular androgen levels (Fig. 5c). Additionally, inhibiting cholesterol synthesis with mevastatin also blunted the PLC-mediated androgen elevation (Fig. 5d). These results demonstrate that P-CoA enhances de novo androgen synthesis via SREBP2 activation and cholesterol biosynthesis.

Given that de novo androgen synthesis is a known driver of castration resistance in PCa, we tested whether P-CoA–mediated androgen production contributes to resistance under androgen-deprived conditions. In LNCaP cells, androgen withdrawal via charcoal-stripped FBS suppressed proliferation, but PLC supplementation partially rescued cell growth (Fig. 5e). PLC also restored expression of prostate-specific antigen (PSA), a canonical AR target gene (Fig. 5f). Similarly, this effect was recapitulated in mouse-derived prostate organoids cultured in DHT-depleted medium, where PLC restored growth (Fig. 5g). Together, these results indicate that P-CoA-induced steroidogenesis can maintain AR signalling and support organoid growth under androgen-deprivation conditions.

To determine whether this mechanism depends on the conversion of cholesterol into steroid hormones, we knocked down *CYP11A1*, which encodes the enzyme that catalyses the first committed step of steroidogenesis by converting cholesterol to pregnenolone (Extended Data Fig. 6d). To verify that the effect of PLC on steroidogenesis is mediated through CYP11A1, we performed ^13^C-cholesterol tracing to monitor pregnenolone synthesis. PLC treatment increased the ratio of ^13^C-pregnenolone to ^13^C-cholesterol, indicating enhanced conversion of cholesterol into pregnenolone, whereas this effect was abolished by *CYP11A1* knockdown (Fig. 5h). CYP11A1 depletion also blocked the PLC-induced increase in intracellular androgen levels and AR activity (Extended Data Fig. 6e,f), restored sensitivity to androgen deprivation (Fig. 5i), and suppressed PLC-induced spheroid expansion and colony formation (Fig. 5j,k). Consistently, pharmacological inhibition of CYP11A1 with ODM-208 likewise suppressed PLC-induced spheroid expansion and colony formation (Extended Data Fig. 6g,h). Together, these results demonstrate that cholesterol-derived steroidogenesis is required for the pro-growth effects of P-CoA.

Castration resistance remains a major clinical challenge in PCa^2^. Given that P-CoA elevation promotes steroidogenesis and AR reactivation, we hypothesized that tumours might upregulate P-CoA in response to AR inhibition as an adaptive mechanism. Supporting this, we found that culturing LNCaP cells in charcoal-stripped FBS significantly increased both intracellular PLC and P-CoA levels (Extended Data Fig. 6i). Similarly, DHT withdrawal elevated PLC in prostate organoids (Extended Data Fig. 6j). Reanalysis of published metabolomic data from patient-derived xenograft models also showed increased PLC levels in tumours following castration (Extended Data Fig. 6k)^29^. Notably, C4-2 cells, derived from castration-resistant LNCaP xenografts, exhibited higher PLC levels than parental LNCaP cells, with an even greater increase observed in C4-2B cells, which originate from bone metastatic sites of C4-2 xenografts (Extended Data Fig. 6l).

To test whether targeting P-CoA biosynthesis could resensitize CRPC cells to androgen deprivation, we knocked down *ALDH6A1* and *ACAT1* to inhibit P-CoA production from isoleucine and valine. This sensitized C4-2 cells to androgen deprivation, an effect that could be partially rescued by PLC supplementation (Fig. 5l). Notably, C4-2 and C4-2B cells were more sensitive than LNCaP cells to isoleucine and valine restriction (Fig. 5m), indicating greater sensitivity to isoleucine and valine restriction in these castration-resistant LNCaP-derived cell lines. Collectively, these results suggest that in response to androgen deprivation, PCa cells increase P-CoA level to sustain cholesterol availability, drive de novo androgen synthesis and maintain AR signalling, thereby facilitating castration resistance and tumour progression.

### Propionyl-CoA-mediated AR signalling activation enhances neutral lipid accumulation

A noteworthy observation from our studies was that short-term PLC treatment produced a durable effect on spheroid and tumour growth (Fig. 1d,g). This suggested that transient elevations in P-CoA may serve as signalling events that trigger downstream metabolic adaptations promoting aggressive tumour phenotypes. Given that cholesterol synthesis and accumulation is a primary response to the elevation of P-CoA, we next investigated the long-term effects of P-CoA elevation on lipid metabolism.

Lipidomic profiling of LNCaP cells treated with LC or PLC for 7 days revealed a broader increase in neutral lipid species, including triglycerides (TGs) and cholesteryl esters, compared with the 3-day treatment (Supplementary Table 1). Neutral lipids are typically sequestered in lipid droplets to mitigate lipotoxicity. Thus, this shift indicates that tumour cells may form more lipid droplets to buffer excess cholesterol and maintain a storage reservoir. Elevated neutral lipids are strongly associated with PCa aggressiveness. Notably, high-grade PCa and metastases exhibit abnormal accumulation of esterified cholesterol in lipid droplets, a feature not seen in benign prostate tissue or prostatic intraepithelial neoplasia lesions^30^. Multiple studies have shown that these lipid droplets serve as cholesterol reserves for steroidogenesis, including androgen production, which fuels AR signalling and PCa progression^31^.

**Fig. 6:**
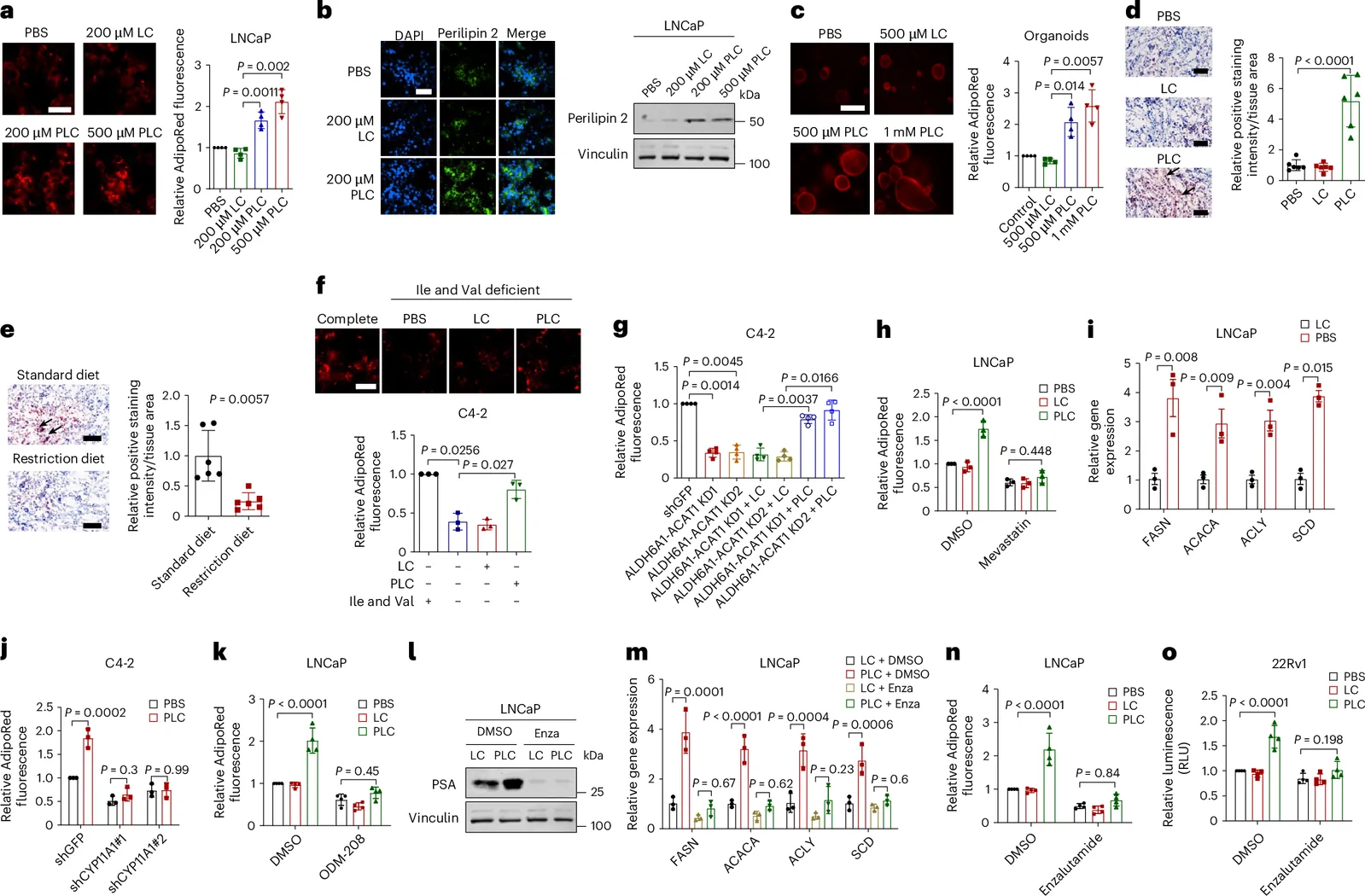
P-CoA-mediated activation of AR signalling enhances neutral lipid accumulation. **a**,**b**, LNCaP cells were treated with LC or PLC for 7 days. Neutral lipid levels were quantified by AdipoRed staining (**a**; *n* = 4), and perilipin 2 levels were assessed by immunofluorescence staining and western blotting (**b**). **c**, Mouse prostate organoids were treated with LC or PLC for 7 days, and neutral lipid levels were quantified and normalized to organoid biomass (*n* = 4). **d**,**e**, Neutral lipid content was assessed in xenograft tumours derived from LNCaP cells pretreated with PBS, LC, or PLC (**d**; *n* = 6 tumours from 6 mice per group) and in tumours from mice fed either a complete control diet or an isoleucine- and valine-restricted diet (**e**; *n* = 6 tumours from 6 mice per group). **f**,**g**, Neutral lipid levels were measured in PCa cells cultured in isoleucine- and valine-deficient medium supplemented with LC or PLC (**f**; *n* = 3), or following simultaneous knockdown of *ALDH6A1* and *ACAT1* and treatment with LC or PLC (**g**; *n* = 4). **h**, PCa cells were treated with LC or PLC, with or without mevastatin, for 7 days, and neutral lipid accumulation was quantified (*n* = 3). **i**, Tumour cells were treated with LC or PLC for 5 days, and mRNA levels of fatty acid synthesis-related genes were measured (*n* = 3). **j**,**k**, Neutral lipid levels were assessed after *CYP11A1* knockdown in C4-2 cells (**j**; *n* = 3) or after ODM-208 treatment in LNCaP cells (**k**; *n* = 3), together with LC or PLC. **l**–**n**, LNCaP cells were treated with LC or PLC for 2 days and then exposed to enzalutamide. PSA protein levels were measured after 3 days (**l**), and mRNA levels of fatty acid synthesis genes were quantified (**m**; *n* = 3). Neutral lipid levels were assessed after 5 days (**n**; *n* = 4). **o**, 22Rv1 cells were treated with LC or PLC for 2 days, followed by enzalutamide for 3 days, and then grown as spheroids for 2 weeks. Spheroid biomass was measured (*n* = 4). Data are presented as mean ± s.d. from independent replicates. Statistical significance was assessed using two-tailed unpaired *t*-test (**e**,**i**), one-way ANOVA (**a**,**c**,**d**,**f**,**g**) and two-way ANOVA (**h**,**j**,**k**,**m**–**o**). Scale bars, 100 μm (**a**,**b**,**d**–**f**), 300 μm (**c**). See also Extended Data Fig. 7. Source data

**Extended Data Fig. 7:**
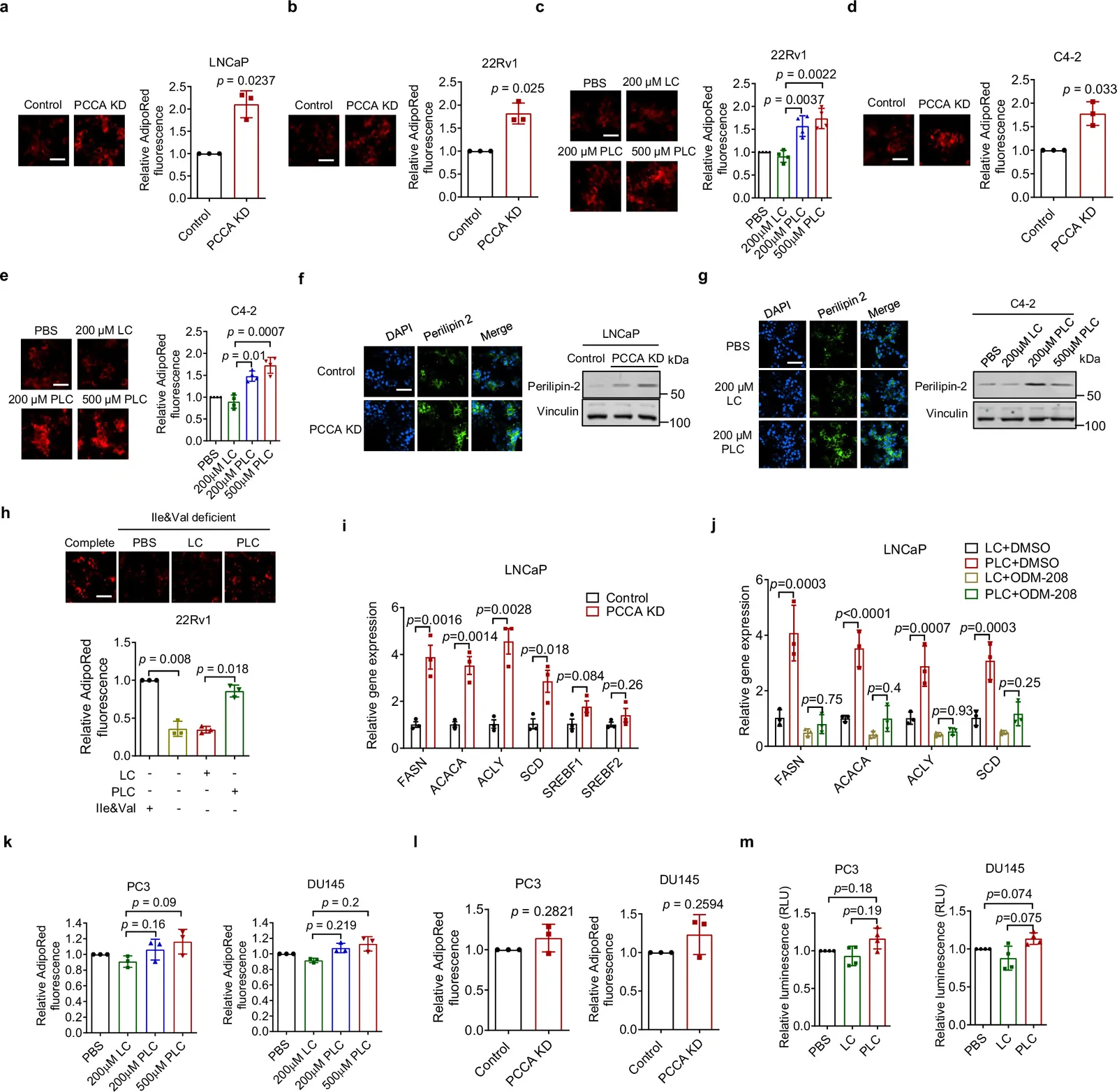
AR-dependent fatty acid synthesis enhances P-CoA-induced neutral lipid accumulation. **a**, LNCaP cells were transfected with siRNA targeting PCCA for 5 days, and neutral lipid levels were assessed by AdipoRed staining. Representative images are shown, and staining intensity was quantified (*n* = 3). **b-e**, PCCA was knocked down, or cells were treated with LC or PLC in 22Rv1 (**b, c**) and C4-2 (**d, e**) cells. Neutral lipid levels were measured 5 days post-knockdown (*n* = 3) or after 7 days of metabolite treatment (*n* = 4). **f**, Following 5 days of PCCA knockdown, perilipin 2 levels were assessed by immunofluorescence staining and western blotting. Perilipin 2 is shown in green, and nuclei were counterstained with DAPI (blue). **g**, Cells were treated with LC or PLC for 7 days, and perilipin 2 levels were assessed by immunofluorescence staining and western blotting. Perilipin 2 is shown in green, and nuclei were counterstained with DAPI (blue). **h**, Cells were cultured in isoleucine- and valine-deficient medium supplemented with 200 μM LC or PLC for 5 days, and neutral lipid levels were assessed by AdipoRed staining (*n* = 3). **i**, mRNA levels of genes associated with fatty acid synthesis were measured in LNCaP cells 5 days after PCCA knockdown (*n* = 3). **j**, LNCaP cells were treated with LC or PLC, with or without ODM-208, for 5 days, and mRNA levels of genes involved in fatty acid synthesis were measured (*n* = 3). **k**, Tumour cells were treated with LC or PLC for 7 days, and neutral lipid content was measured (*n* = 3). **l**, Cells were transfected with siRNA targeting PCCA or a non-targeting control siRNA for 5 days, followed by quantification of neutral lipid content. **m**, Cells were pretreated with LC or PLC for 5 days and then seeded under spheroid-forming conditions. Spheroid biomass was quantified using CellTiter-Glo (*n* = 4). Data are presented as mea*n* ± s.d. from independent replicates. The western blots shown are representative of three independent experiments with similar results. Statistical significance was assessed using two-tailed paired t-test (**a, b, d, i, l**); two-way ANOVA (**j**); and one-way ANOVA (**c, e, h, k, m**). Scale bars: **a-h**, 100 μm. Source data

To directly assess neutral lipid levels, we employed AdipoRed staining. Both *PCCA* knockdown and PLC treatment significantly increased intracellular neutral lipid accumulation in LNCaP cells (Fig. 6a and Extended Data Fig. 7a). Similar effects were observed in AR-positive, castration-resistant PCa cell lines 22Rv1 and C4-2 (Extended Data Fig. 7b–e). As neutral lipids are stored in lipid droplets coated by perilipins, we measured perilipin 2 levels and found that they were elevated following *PCCA* knockdown or PLC treatment (Fig. 6b and Extended Data Fig. 7f–g), corroborating increased lipid droplet formation. Consistently, PLC supplementation also enhanced neutral lipid accumulation in mouse prostate organoids (Fig. 6c). In vivo, tumours derived from PLC-pretreated cells exhibited significantly higher neutral lipid levels compared with controls (Fig. 6d).

As isoleucine and valine catabolism is a primary source of P-CoA, we next examined whether restricting these amino acids could suppress lipid buildup. Mice on an isoleucine/valine-restricted diet exhibited reduced tumour PLC levels and corresponding decreases in neutral lipid content (Fig. 6e). This effect was reproduced in vitro: isoleucine and valine deprivation decreased neutral lipid content, which was restored by PLC supplementation (Fig. 6f and Extended Data Fig. 7h). Knockdown of *ALDH6A1* and *ACAT1* also reduced neutral lipid accumulation, which could again be partially rescued by PLC (Fig. 6g), indicating that isoleucine/valine regulate lipid storage through P-CoA generation.

Next, we investigated the mechanism linking early cholesterol synthesis to downstream neutral lipid storage. Blocking cholesterol synthesis with mevastatin abolished PLC-induced neutral lipid accumulation (Fig. 6h), indicating that de novo cholesterol biosynthesis is a prerequisite for the observed lipid buildup. As lipid droplets commonly store cholesteryl esters and TGs, we next asked how tumour cells generate sufficient fatty acid substrates in response to elevated P-CoA. P-CoA elevation upregulated genes involved in fatty acid biosynthesis at later time points, supporting a coordinated anabolic programme (Fig. 6i and Extended Data Fig. 7i).

We then asked whether cholesterol elevation alone was sufficient to drive this response, or whether it required further signalling input. Given AR’s central role in regulating lipid metabolism in PCa and the ability of P-CoA to enhance steroidogenesis, we posited that AR activation might mediate the observed lipid accumulation. Supporting this, knockdown of *CYP11A1* attenuated androgen synthesis and AR activity (Extended Data Fig. 6d–f) and also blocked PLC-induced neutral lipid accumulation (Fig. 6j). Pharmacological inhibition of CYP11A1 using ODM-208 produced similar effects, preventing both lipid accumulation and the induction of lipogenic genes (Fig. 6k and Extended Data Fig. 7j). These findings suggest that cholesterol elevation alone is insufficient; activation of AR signalling is required to initiate the downstream lipogenic programme.

To further confirm AR’s role, we treated LNCaP cells with enzalutamide to block AR activity (Fig. 6l). Enzalutamide abolished PLC-induced upregulation of lipogenic genes and suppressed neutral lipid accumulation (Fig. 6m,n). As long-term AR inhibition suppresses LNCaP cell proliferation, we turned to 22Rv1 cells, which harbour both full-length and truncated AR variants, to assess the impact of AR blockade on spheroid growth. Enzalutamide significantly impaired PLC-induced spheroid expansion in 22Rv1 cells (Fig. 6o), further supporting AR’s role in mediating this effect. Finally, we tested whether this mechanism is dependent on AR by comparing AR-negative PCa cell lines (PC3 and DU145). In these cells, P-CoA elevation had only modest effects on neutral lipid accumulation and spheroid growth (Extended Data Fig. 7k–m), providing additional evidence consistent with an AR-dependent contribution.

**Fig. 7:**
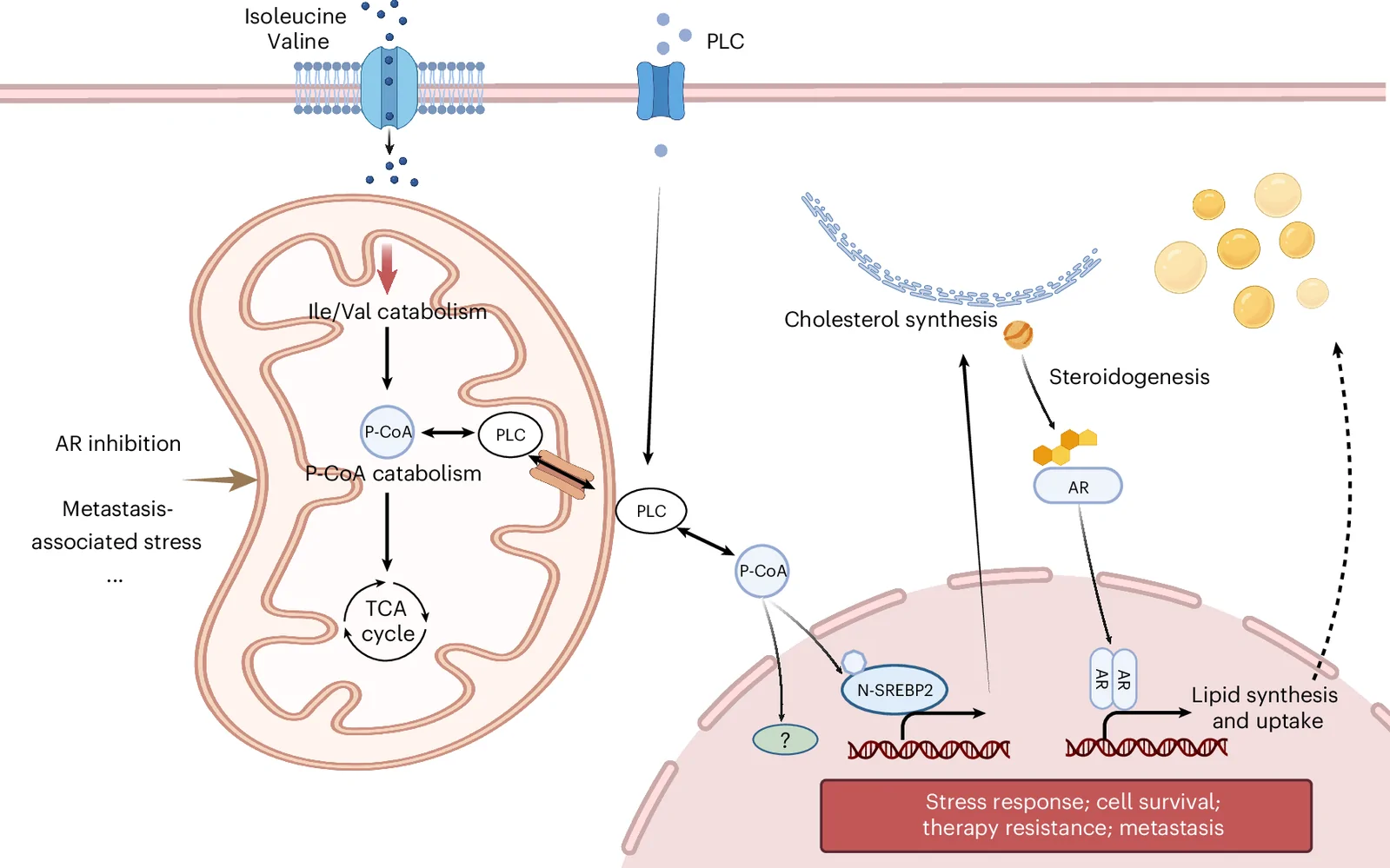
Proposed model of P-CoA-mediated lipid metabolic reprogramming and castration resistance in prostate cancer. In response to therapy-induced or metastasis-associated stress, PCa cells increase P-CoA and PLC levels by enhancing isoleucine and valine catabolism and/or suppressing P-CoA degradation. Elevated P-CoA functions as a signalling metabolite that promotes lysine propionylation and stabilization of nuclear SREBP2. Stabilized SREBP2 enhances cholesterol biosynthesis and increases the storage of cholesterol and cholesteryl esters in lipid droplets. These cholesterol pools can subsequently support de novo steroidogenesis, including androgen production, thereby activating AR signalling. In turn, AR signalling promotes fatty acid synthesis and lipid uptake, further enhancing neutral lipid accumulation. This feedforward circuit supports PCa cell survival and proliferation under androgen-deprived conditions and may contribute to castration resistance and disease progression.

In summary, P-CoA functions as a metabolic signal that initiates cholesterol synthesis, steroidogenesis and AR activation. This, in turn, drives fatty acid synthesis and neutral lipid accumulation via a feedforward loop. This adaptive mechanism enables PCa cells to withstand metabolic stress and androgen deprivation, ultimately contributing to disease progression (Fig. 7).

## Discussion

Unlike normal cells, which primarily rely on extracellular lipids, many cancer cells stimulate de novo lipid synthesis to meet their metabolic demands^32^. Precise regulation of cholesterol dynamics is critical for tumour survival and progression^7^. Yet, how tumour cells dynamically regulate cholesterol synthesis, particularly how they overcome feedback inhibition by cholesterol, remains unclear. In this study, we uncover a previously unrecognized regulatory mechanism by which prostate tumour cells enhance cholesterol synthesis, accumulate lipid droplets, and sustain AR signalling to better adapt to stress and support tumour progression.

Beyond its structural role in membranes, cholesterol is also the primary substrate for steroidogenesis. Cholesterol metabolism is tightly linked to stress response pathways, especially in hormone-sensitive cancers such as PCa. While steroidogenesis typically occurs in specialized tissues like gonads and adrenal glands, it becomes aberrantly activated during prostate tumorigenesis and progression. The extent of this activity contributes to castration resistance and modulates dependence on exogenous androgens in AR-positive tumour cells. To avoid cytotoxicity, excess cholesterol is esterified and stored in lipid droplets. Notably, enzymes such as sterol O-acyltransferase 1 (SOAT1), which mediate cholesterol esterification, are generally highly expressed in prostate tumours, enabling the safe storage of cholesterol while maintaining a reservoir readily available for steroidogenesis. Although cholesterol likely contributes to cancer progression through multiple mechanisms, including membrane dynamics and signalling pathways, these aspects warrant future investigation.

Effective stress adaptation is crucial for tumour cells to survive environmental challenges such as overgrowth, metastasis and therapeutic pressure^33^. During this process, tumours often acquire aggressive features, including stem-like properties. We observed evidence of enhanced P-CoA/PLC metabolism in advanced disease and increased P-CoA availability in response to androgen deprivation, both conditions associated with stress-induced reprogramming into stem-like states. The phenotypes associated with P-CoA elevation, including increased neutral lipid accumulation, enhanced metastatic potential and castration resistance, are hallmark features often associated with cancer stemness^34^. Of note, the tumour-promoting effects of P-CoA were more pronounced in 3D cultures than in 2D, suggesting that P-CoA-mediated lipid accumulation plays a particularly important role under the heightened stress conditions imposed by 3D growth. Moreover, P-CoA-driven androgen synthesis may help sustain AR signalling, which is typically reduced in 3D cultures. In contrast, excessive AR signalling in 2D conditions can paradoxically suppress proliferation. Inhibition of steroidogenesis effectively blocked P-CoA-induced spheroid growth and colony formation, indicating that both enhanced AR signalling and increased lipid accumulation contribute to the adaptive response.

A critical yet unresolved question is how stress, such as androgen deprivation, upregulates P-CoA. Our transcriptomic comparison between primary hormone-sensitive and metastatic castration-resistant prostate tumours suggests a dual mechanism involving increased upstream production and decreased catabolism of P-CoA. For instance, the downregulation of *PCCA* alone can elevate intracellular P-CoA levels. However, prolonged *PCCA* knockout also induces cell stress and senescence-like phenotypes, likely due to excessive P-CoA accumulation and metabolic toxicity, resembling the pathology of propionic acidaemia. These observations support the idea that P-CoA levels are dynamically regulated as part of a stress-adaptive response, and artificial manipulation that overdrives this pathway may overwhelm the cell’s adaptive capacity and prove toxic.

Another major finding of our study is the identification of SREBP2 as a downstream target of P-CoA. Previous studies have established a positive feedback loop between AR signalling and SREBPs, wherein AR promotes SREBP activation, and SREBPs enhance AR expression and lipid metabolism^35,36^. This explains why P-CoA’s effects on cholesterol metabolism are more pronounced in AR-positive tumours. Notably, while active SREBP1 remains stable, SREBP2 activity increases during progression and post-castration^37^. We found that P-CoA specifically modifies SREBP2, but not SREBP1, likely due to structural differences between the isoforms that confer selective susceptibility to lysine propionylation^38^. This selective modification may underlie the preferential activation of SREBP2 in advanced disease. Notably, we identified KAT3A and KAT3B as the candidate enzymes mediating SREBP2 propionylation. Interestingly, these lysine acetyltransferases have previously been implicated as key drivers of PCa aggressiveness^39^. Their ability to catalyse SREBP2 propionylation may represent a new mechanism by which they promote tumour progression. Although SREBP2 is traditionally associated with regulating cholesterol synthesis and uptake, our data reveal that propionylated SREBP2 preferentially enhances cholesterol synthesis, suggesting that P-CoA-SREBP2 signalling is context-dependent and may also involve histone propionylation, further modulating gene expression in a locus-specific manner^40^.

Of note, the P-CoA-induced activation of SREBP2 circumvents the classical feedback inhibition by cholesterol, allowing sustained cholesterol synthesis despite rising intracellular cholesterol levels. Although SREBP2 is a key effector in this model, other propionylation targets may contribute to this regulatory programme. Statins, which inhibit cholesterol synthesis, have shown mixed results in PCa treatment^41,42^. Our findings suggest that patients with tumours driven by P-CoA elevation might benefit more from statin therapy. More notably, targeting P-CoA or its effect on SREBP2 may provide a broader therapeutic approach, as it could simultaneously suppress cholesterol synthesis and other oncogenic pathways, such as MYC^43^, offering a broader impact on tumour progression.

BCAAs constitute a substantial portion of dietary and essential amino acids and have emerged as key metabolic regulators beyond their traditional role in protein synthesis. In cancer patients, cancer-associated cachexia, marked by progressive muscle and adipose tissue wasting, may contribute to elevated circulating BCAA levels, thereby supplying tumour cells with abundant substrates to engage this metabolic pathway and promote aggressive behaviour. Numerous studies have reported elevated serum BCAA levels in various models of metabolic disease, implicating them in disease progression^44^. While BCAAs have been traditionally linked to lipid metabolism through their contribution to the TCA cycle^45^, our study reveals a distinct regulatory role for isoleucine and valine in lipid synthesis via P-CoA production, establishing a functional link between mitochondrial BCAA catabolism and nuclear signalling through P-CoA-mediated protein modification. Previous studies have shown that isoleucine is a major contributor to P-CoA production in multiple cell types, with valine playing a more limited role^14^. In contrast, our PCa model reveals that both isoleucine and valine significantly contribute to P-CoA biosynthesis. Notably, leucine, which robustly activates mTOR signalling, did not rescue the lipid synthesis defects caused by isoleucine and valine restriction, highlighting a mechanistically distinct role for these BCAAs. Consistent with this, recent work demonstrates that isoleucine and valine regulate lipid metabolism independently of leucine. Moreover, dietary restriction of isoleucine and valine has been shown to improve metabolic health and reduce lipid accumulation in vivo^46^. The regulatory axis uncovered in our model may help explain the metabolic benefits observed in these dietary interventions.

Although our findings may have broader relevance beyond PCa, the downstream effects of P-CoA signalling are likely context-dependent. The role of cholesterol in tumour growth, the regulatory mechanisms controlling cholesterol homeostasis, the dependency on steroid hormones, and the activity of the propionate metabolism pathway all vary between tumour types and may influence the biological outcomes of P-CoA elevation. Notably, enzymes such as PCCA are more highly expressed in PCa compared with other malignancies^47^. Moreover, a cancer’s reliance on lipid metabolism may influence how P-CoA shapes tumour behaviour. For instance, in hepatocellular carcinoma, P-CoA exerts an antiproliferative effect by disrupting citrate synthase activity and impairing the TCA cycle^48^. These tissue-specific differences likely stem from divergent expression and activity of SREBPs and their downstream metabolic programmes, highlighting the importance of cellular context in shaping the metabolic impact of P-CoA.

In conclusion, this study expands our understanding of the multifaceted roles of P-CoA in prostate cancer progression, particularly during early adaptation to androgen deprivation. Our findings indicate that elevated P-CoA promotes cholesterol biosynthesis, supports cholesterol-dependent steroidogenesis, and contributes to resistance to AR-targeted therapies, processes that may also facilitate subsequent disease progression. Although current therapeutic approaches target cholesterol or steroid synthesis, strategies that limit P-CoA production or disrupt its downstream activation of SREBP2, including interventions targeting isoleucine and valine metabolism, may provide an alternative means of suppressing this metabolic programme. These findings support further investigation of P-CoA metabolism as a potential therapeutic vulnerability in prostate cancer and, more broadly, in other malignancies that depend on lipid metabolic reprogramming.

## Methods

### Animals

Male athymic nude (nu/nu) mice (7–8 weeks old) were obtained from Envigo and acclimated for 7–10 days at the Weill Cornell Medicine Belfer Research Building Vivarium. Mice were housed under standard conditions (≤5 per cage) with ad libitum access to acidified water and PicoLab Rodent Diet 5053 (20% protein, 5% fat). Husbandry followed Animal Biosafety Level 2 procedures in a xenograft-designated area, with environmental conditions maintained at 21–23 °C on a 12-h light–dark cycle. For amino acid-restriction studies, isoleucine and valine levels were reduced while total amino acid content was maintained by increasing other amino acids (Supplementary Table 2).

Tumour cells were transduced with an EF1α-luciferase-P2A-GFP vector, then trypsinized and resuspended in a 1:1 mixture of PBS and Matrigel. For subcutaneous xenografts, 1 × 10^6^ cells in 100 μl were injected into the left flank. For lung colonization, 2 × 10^6^ cells in 100 μl serum-free RPMI were injected via the tail vein. After engraftment, mice received either the isoleucine- and valine-restricted diet or a control diet. Tumour growth and lung colonization were monitored weekly using an IVIS Spectrum CT system, with bioluminescence quantified using Living Image software (v.4.5). Signals were normalized to the first measurement at 1 week post-engraftment.

Mice were randomly assigned to groups (up to ten per group) and no blinding was applied during the study. No animals were excluded unless noted. All procedures were approved by the Weill Cornell Medicine Institutional Animal Care and Use Committee (protocol no. 2014-0060). Humane end points included a maximum tumour diameter of 20 mm, volume of 2.5 cm^3^, or tumour burden exceeding 10% of body weight.

### Cell culture

LNCaP (CRL-1740), C4-2 (CRL-3314), C4-2B (CRL-3315), 22Rv1 (CRL-2505), PC3 (CRL-1435) and DU145 (HTB-81) PCa cell lines were purchased from ATCC and cultured in RPMI-1640 medium (Corning) supplemented with 10% FBS (Sigma-Aldrich) and 1% penicillin–streptomycin (Gibco). LNCaP cells were cultured on poly-L-lysine (Sigma-Aldrich, P1274) coated plates. HEK293T (Q401) cells were obtained from GenHunter and cultured in high-glucose DMEM (Gibco) with 10% FBS and 1% penicillin–streptomycin. To remove different components from the FBS in the cell culture, the medium was supplemented with 10% dialysed FBS, 10% lipoprotein-deficient FBS (Alfa Aesar) or 1% fatty-acid-free BSA (Sigma-Aldrich). To deplete steroid hormones from the medium, the tumour cells were cultured in 10% charcoal/dextran-treated FBS (Omega Scientific, FB-04). All cell lines were maintained at 37 °C in a humidified atmosphere with 5% CO_2_. Routine testing confirmed that all cell lines were *Mycoplasma*-free.

### Organoid culture

Mouse prostate organoids with SPOP mutation, AR positivity, and androgen dependency were obtained from the Barbieri laboratory^49^. The organoids were cultured and passaged as previously described^50^. To establish androgen-deprived conditions, DHT was removed from the culture medium. Organoid biomass was quantified using the CellTiter-Glo 3D Cell Viability Assay (Promega, G9683).

### Medium nutrient manipulation

Threonine, methionine and branched-chain amino acids were obtained from Sigma-Aldrich. LC, PLC and acetyl-L-carnitine were obtained from Cayman Chemical Company. Succinyl-L-carnitine was obtained from Santa Cruz Biotechnology. All carnitine derivatives were dissolved in PBS. For BCAA manipulation, RPMI-1640 medium devoid of BCAAs was custom prepared by the Memorial Sloan Kettering Cancer Center (MSKCC) Medium Center and used as the base medium. To establish specific BCAA deprivation conditions, individual BCAAs were reintroduced as required. Similarly, threonine- and methionine-deficient RPMI medium was prepared by MSKCC, with threonine or methionine added back to create selective deficiency conditions.

For experiments aimed at downregulating P-CoA through restriction of isoleucine and valine, leucine was reintroduced to maintain total BCAA levels. To minimize interference from amino acids present in serum, the medium was supplemented with 10% dialysed FBS (dFBS). The culture medium was replaced daily with fresh medium containing the indicated metabolites. The concentrations of metabolites used in individual experiments are provided in the corresponding figure legends.

### Drug treatment

Mevastatin, 25-hydroxycholesterol (25-HC), betulin, PF-429242, opevesostat (ODM-208) and enzalutamide were dissolved in dimethylsulfoxide, aliquoted, and stored at −20 °C. Fresh aliquots were thawed immediately before treatment. The following final concentrations were used: mevastatin (500 nM, Cayman Chemical, 10010340), 25-HC (5 µM, Selleck Chemicals, S8091), betulin (2 µM, MedChemExpress, HY-N0083), PF-429242 (10 µM, Cayman Chemical, 15140), ODM-208 (10 µM, Selleck Chemicals, E1650) and enzalutamide (40 µM, Selleck Chemicals, S1250). Dimethylsulfoxide at the same volume was used as a control. Treatments were applied after cell attachment to the plates.

### Neutral lipid staining

For live imaging, tumour cells or organoids were pretreated with metabolites or inhibitors and then seeded onto glass-bottom dishes (Cellvis, D29-14-1.5-N). The same treatment conditions were maintained until an appropriate cell density was reached. The culture medium was then removed, and the cells or organoids were washed with PBS. AdipoRed reagent (Lonza, PT-7009) was diluted 1:50 in PBS, added to the dishes, and incubated for 10 min. Fluorescence images were acquired using a fluorescence microscope.

For quantification, pretreated cells or organoids were seeded in 96-well plates (opaque walls and clear bottoms) and maintained under the same treatment conditions. AdipoRed reagent was added (100 μl per well) and incubated for 10 min. Fluorescence was measured (Ex 485 nm/Em 572 nm). Wells without cells or with Matrigel alone served as background controls. Biomass was measured in parallel plates using crystal violet staining (tumour cells) or CellTiter-Glo 3D (organoids), and AdipoRed fluorescence was normalized accordingly.

For tissue staining, xenograft tumours were collected, washed with PBS, fixed in 10% formalin for 24 h, and cryoprotected in 30% sucrose. Samples were embedded, frozen and sectioned (10–12 μm). Sections were equilibrated in 60% isopropanol, stained with Oil Red O for 10–15 min, rinsed, counterstained with hematoxylin, and mounted. Slides were scanned using a ZEISS Axioscan 7, and staining was quantified in Fiji/ImageJ by measuring red signal intensity or area.

### Immunofluorescent staining

After pretreatment, tumour cells were cultured on glass slides in 12-well plates. When cell density reached 80%, cells were fixed with 4% formaldehyde for 15 min at room temperature and washed with PBS. Cells were permeabilized with 0.04% Triton X-100 for 30 min and then washed with PBS. Nonspecific binding was blocked using LI-COR blocking buffer for 2 h at room temperature. A rabbit anti-perilipin 2 primary antibody (Cell Signalling Technology) was diluted 1:200 in LI-COR blocking buffer and incubated with the cells for 3 h at room temperature. Cells were washed three times with PBS and then incubated with an Alexa Fluor 488-conjugated anti-rabbit secondary antibody for 1 h at room temperature. After five washes with PBS, coverslips were mounted using Dako Fluorescence Mounting Medium containing 4,6-diamidino-2-phenylindole (DAPI; Agilent Technologies). Images were acquired using a Nikon fluorescence microscope.

### Immunohistochemistry staining

Resected xenograft tumours were fixed overnight in 10% neutral-buffered formalin at 4 °C, dehydrated and embedded in paraffin. Paraffin-embedded tissues were sectioned at a thickness of 5 μm and mounted onto glass slides. Sections were deparaffinized in xylene, rehydrated through a graded ethanol series, and rinsed in PBS. For antigen retrieval, sections were heated in 10 mM citrate buffer (pH 6.0) using a microwave at medium power for 15 min and then allowed to cool to room temperature. Endogenous peroxidase activity was blocked by incubation with 3% hydrogen peroxide in methanol for 10 min. To reduce nonspecific binding, sections were incubated for 1 h at room temperature in PBS containing 5% normal serum from the host species of the secondary antibody.

Sections were incubated overnight at 4 °C in a humidified chamber with an anti-Ki-67 primary antibody (Cell Signalling Technology). After washing with PBS, sections were incubated with the appropriate biotinylated secondary antibody for 1 h at room temperature, followed by incubation with an avidin–biotin complex reagent (Vector Laboratories) for 30 min. Immunoreactivity was visualized using a diaminobenzidine substrate, and sections were counterstained with hematoxylin, dehydrated and mounted.

Ki-67-positive cells were identified by brown nuclear staining. The percentage of Ki-67-positive cells was determined by counting positively stained and total cells in five randomly selected high-power fields per tumour at ×400 magnification using Fiji/ImageJ. Image selection and quantification were performed with the investigator blinded to the experimental groups.

### Colony-formation assays

The base layer of agarose gel plates was prepared by mixing equal volumes of 1.2% agarose and 2× culture medium containing 20% FBS, resulting in final concentrations of 0.6% agarose and 10% FBS. The mixture was added to the culture plates and allowed to solidify at room temperature.

For the upper layer, 5,000 cells were suspended in a mixture containing equal volumes of 0.6% agarose and 2× culture medium supplemented with 20% FBS, resulting in final concentrations of 0.3% agarose and 10% FBS. After the upper layer solidified, the plates were transferred to a cell-culture incubator. One millilitre of culture medium containing the indicated treatments was added above the agarose layer. The overlay medium was replaced every 2 days while maintaining the same treatment conditions. After approximately 3 weeks, colonies were stained with 0.1% crystal violet prepared in 10% methanol for 10 min and then rinsed five times with distilled water. Plates were scanned, and colonies were counted using Fiji/ImageJ.

### Spheroid growth assays

Following the indicated pretreatments or genetic manipulations, 5,000 cells were seeded per well in ultra-low-attachment 96-well U-bottom plates (S-Bio, MS-9096UZ) in 100 μl of culture medium. After 5 days, 50 μl of fresh medium was added to each well. Subsequently, 50 μl of medium was carefully removed and replaced with fresh medium every 3 days while maintaining the indicated treatment conditions. After 2 weeks, spheroid biomass was quantified using the CellTiter-Glo 3D Cell Viability Assay according to the manufacturer’s instructions.

### Immunoblotting and immunoprecipitation

Proteins were extracted using RIPA buffer (40 mM HEPES, pH 7.4, 1 mM EDTA, 120 mM NaCl, 0.5 mM dithiothreitol (DTT), 10 mM β-glycerophosphate, 1 mM NaF, 1 mM Na_3_VO_4_, 0.1% Brij-35, 0.1% deoxycholate and 0.5% NP-40) supplemented with protease inhibitors (1 mM phenylmethyl sulfonyl fluoride (PMSF), 5 µg ml^−1^ pepstatin A, 10 µg ml^−1^ leupeptin and 5 µg ml^−1^ aprotinin). The lysates were incubated on ice for 15 min, followed by incubation with 4× LDS buffer for 5 min at 95 °C. Nuclear and cytoplasmic protein fractions were isolated by using NE-PER Nuclear and Cytoplasmic Extraction Reagents (Thermo Fisher Scientific, 78833). Protein concentrations were determined using the DC Protein Assay kit II (Bio-Rad). After protein extraction and quantification, 2 mg of total protein was used for each immunoprecipitation (IP) reaction. Anti-FLAG M2 affinity gel beads (Sigma-Aldrich, A2220) were pre-washed three times with protein lysis buffer to remove storage buffer and equilibrate the beads. Protein lysates were incubated with 40 μl of washed FLAG beads at 4 °C for 2 h with gentle rotation. Following incubation, the beads were washed three times with ice-cold TBS to remove unbound proteins. Bound proteins were then eluted by adding Laemmli sample buffer directly to the beads and heating at 95 °C for 5 min.

Equal amounts of protein were separated by SDS–PAGE under reducing conditions and transferred to nitrocellulose membranes (GE Healthcare). Membranes were blocked with TBS-based Odyssey Blocking Buffer (LI-COR) and probed with primary antibodies overnight at 4 °C. The antibodies used in this study are listed in Supplementary Table 3. Following primary antibody incubation, membranes were incubated with donkey anti-rabbit (LI-COR Biosciences, 926-32213) or donkey anti-mouse (LI-COR Biosciences, 926-32212) secondary antibodies for 1 h at room temperature. Signals were developed using the LI-COR Odyssey CLx Imaging System.

### Cycloheximide chase assay and analysis of SREBP2 ubiquitylation

To assess SREBP2 protein stability, cells were cultured to approximately 80–90% confluence and treated with cycloheximide (CHX; Sigma-Aldrich, C4859) at a final concentration of 100 μg ml^−1^ to inhibit de novo protein synthesis. Cells were collected at the indicated time points after CHX addition (0, 1, 2, 3, 6 and 9 h). Total cell proteins were extracted, and nuclear SREBP2 levels were analysed by western blotting.

To assess SREBP2 ubiquitylation, cells were treated with 10 μM MG132 for 4 h before collection. SREBP2 was immunoprecipitated, and its ubiquitylation was assessed by western blotting using an anti-ubiquitin antibody (58395, Cell Signalling Technology, 1:1,000).

### Quantitative PCR

RNA was extracted from cells using the PureLink RNA Mini kit (Ambion/Life Technologies) according to the manufacturer’s instructions and treated with DNase I (amplification grade, Sigma-Aldrich). Complementary DNA was synthesized using the iScript cDNA Synthesis kit (Bio-Rad). Quantitative PCR was performed using SYBR Green Master Mix (Life Technologies) on a QuantStudio 6 Real-Time PCR System, and data were collected using QuantStudio Real-Time PCR Software, v.1.3 (Life Technologies). Exported data were further processed using Microsoft Excel. Target gene expression was normalized to the geometric mean of *TBP* and *ACTB* expression. Primer sequences are included in Supplementary Table 4.

### Gene silencing

Lentiviral particles were produced by co-transfecting HEK293T cells with the indicated shRNA plasmids together with the packaging plasmid psPAX2 (Addgene plasmid 12260) and the envelope plasmid pMD2.G (Addgene plasmid 12259), using X-tremeGENE HP DNA Transfection Reagent (Roche) according to the manufacturer’s instructions. Medium was replaced 16 h post-transfection. Lentivirus-containing supernatants were collected over 72 h, filtered, and used to infect tumour cells with 8 μg ml^−1^ Polybrene (Sigma-Aldrich). Selection with 2 μg ml^−1^ puromycin (Sigma-Aldrich) was initiated 24 h after transduction. Information on the lentiviral shRNAs used in this study is provided in Supplementary Table 5.

All siRNAs were obtained from Horizon Discovery as SMARTpool reagent. Lyophilized siRNAs were reconstituted in 250 μl RNase-free water to generate 20 μM stock solutions. Cells were transfected with pooled siRNAs at a final concentration of 30 nM using Lipofectamine RNAiMAX Transfection Reagent (Invitrogen, 13778075) according to the manufacturer’s instructions. Catalogue numbers for all siRNAs are included in Supplementary Table 5.

### Gene overexpression, plasmid construction and site-directed mutagenesis

To generate a lentiviral construct expressing FLAG–streptavidin-tagged SREBP2, human *SREBF2* cDNA was amplified by PCR from pLenti6.3-SREBP2-V5 (DNASU, Clone HsCD00954081) using the following primers: forward: 5′-GCGCTCGAGATGGACGACAGCGGCGAGCTG-3′; reverse: 5′-GCGTCTAGAGGAGGCGGCAATGGCAGTGC-3′. PCR amplification was performed using Phusion High-Fidelity DNA Polymerase (Thermo Fisher Scientific, F530L) according to the manufacturer’s instructions.

The Gateway entry vector pENTR-FS-N, a kind gift from the B. Wang laboratory^51^, was engineered by inserting a double tag (Flag and streptavidin, FS) into the pENTR4 (Addgene plasmid #17424). The PCR-amplified *SREBF2* cDNA and pENTR-FS-N vector were digested with XhoI (NEB, R0146S) and XbaI (NEB, R0145S), and ligated to generate pENTR-FS-SREBP2. The FS-SREBP2 insert in the sequence-verified entry clone was transferred into the Gateway-compatible destination vector pLenti-CMV-puro DEST (Addgene, #17452) using LR Clonase II Enzyme Mix (Thermo Fisher Scientific, 11791-100). The resulting pLenti-CMV-puro-FS-SREBP2 construct was sequence-verified before use.

Site-directed mutagenesis was used to generate lysine-to-arginine substitutions at residues K314, K327 and K378 of human SREBP2. Mutagenesis was carried out using the Q5 Site-Directed Mutagenesis kit (New England Biolabs, E0554) following the manufacturer’s protocol. Primer sequences and specific annealing/extension conditions are provided in Supplementary Table 4. Following PCR amplification, kinase–ligase–DpnI treatment was performed to phosphorylate and circularize the amplified product and digest the parental plasmid template. The reaction was incubated at room temperature for 10 min, and 5 μl of the reaction mixture was transformed into IG 5-α chemically competent cells (Intact Genomics, 1031-24). Plasmid DNA was isolated using the QIAprep Spin Miniprep kit (QIAGEN, 27106), and the intended mutations were confirmed by Sanger sequencing. The corresponding FLAG-tagged empty vector was used as a control.

For SREBP1 overexpression, pcDNA3.1-2×FLAG-SREBP1a (Addgene plasmid 26801) and pShuttle-CMV-FLAG-SREBP-1c (Addgene plasmid 237348) were used. For expression of the transcriptionally active form of SREBP2, pcDNA3.1-2×FLAG-SREBP2 (Addgene plasmid 26807) was used. For transient transfection in six-well plates, 5 μg plasmid DNA was transfected into PCa cells using Lipofectamine 2000 (Invitrogen, 11668019) according to the manufacturer’s instructions. The culture medium was replaced the following day, after which the indicated treatments were initiated.

### Metabolite extraction and analysis

Total metabolites were extracted as previously described^16^. In brief, cells cultured in 6-cm dishes were washed twice with ice-cold PBS and ice-cold 80% methanol was added directly to the dishes. Samples were incubated at −80 °C for 30 min and then centrifuged at 21,300*g* for 30 min at 4 °C. The supernatants were transferred to fresh tubes and dried under vacuum using a centrifugal evaporator. For PLC analysis, dried metabolite extracts were resuspended in 60 μl of 50% methanol in water and centrifuged at 21,000*g* for 20 min. The resulting supernatants were analysed using an Agilent 1290 LC system coupled to an Agilent 6530 quadrupole time-of-flight mass spectrometer equipped with a Bio-Bond C4 column (5 μm, 4.6 × 50 mm). Mobile phase A consisted of water containing 0.1% formic acid and 5 mM ammonium formate, and mobile phase B consisted of methanol containing 0.1% formic acid and 5 mM ammonium formate. Samples were injected at 10 μl per run. The flow rate was 0.35 ml min^−1^, and the column temperature was maintained at 40 °C. The mobile phase was held at 50% A for 0–6 min. Peaks were identified by comparison with authentic standards. Metabolite abundance was normalized to the number of cells used for extraction.

### Targeted metabolomics

Targeted metabolomics was performed to characterize serum metabolite changes in mice fed an isoleucine- and valine-restricted diet. Blood was collected by cardiac puncture and allowed to clot at room temperature for 30 min. Samples were then centrifuged at 1,000–2,000*g* for 15 min, and the supernatant was collected as serum. Metabolites were extracted from the serum using pre-chilled 80% methanol stored at −80 °C. Extracts were incubated at −80 °C, dried using a centrifugal evaporator, and reconstituted in HPLC-grade water before analysis by hydrophilic interaction liquid chromatography-mass spectrometry. Metabolites were measured on a Q Exactive Orbitrap mass spectrometer (Thermo Scientific), which was coupled to a Vanquish UPLC system (Thermo Scientific) via an Ion Max ion source with a HESI II probe (Thermo Scientific). A Sequant ZIC-pHILIC column (2.1 mm internal diameter) i.d. × 150 mm, particle size of 5 µm, Millipore Sigma) was used for separation of metabolites. A 2.1 × 20-mm guard column with the same packing material was used for protection of the analytical column. The flow rate was set at 150 μl min^−1^. Buffers consisted of 100% acetonitrile for mobile phase A, and 0.1% NH_4_OH/20 mM CH_3_COONH_4_ in water for mobile phase B. The chromatographic gradient ran from 85% to 30% A in 20 min followed by a wash with 30% A and re-equilibration at 85% A. The Q Exactive was operated in full scan, polarity-switching mode with the following parameters: the spray voltage 3.0 kV, the heated capillary temperature 300 °C, the HESI probe temperature 350 °C, the sheath gas flow 40 units, the auxiliary gas flow 15 units. MS data acquisition was performed in the m/z range of 70–1,000, with 70,000 resolution (at 200 m/z). The AGC target was 1e6 and the maximum injection time was 250 ms. The MS data were processed using XCalibur v.4.1 (Thermo Scientific) to obtain metabolite signal intensities. Identification required exact mass (within 5 ppm) and standard retention times.

### Stable isotope tracing

For stable isotope-tracing experiments, LNCaP cells were seeded in RPMI-1640 medium supplemented with 10% dialysed FBS. After 24 h, cells were washed three times with PBS to remove residual unlabelled nutrients and then incubated in isotope-tracing medium for 24 h. BCAA-tracing medium was prepared using a custom BCAA-depleted RPMI-1640 base medium, supplemented with regular unlabelled leucine (50 mg l^−1^) and either [U-^13^C_6_]-L-isoleucine (50 mg l^−1^; Cayman Chemical, 9004090) or [U-^13^C_5_]-L-valine (20 mg l^−1^; MedChemExpress, HY-N0717S6). Following incubation, cells were collected for metabolite extraction.

Metabolites were extracted using 80% methanol. The extracts were dried down and then re-dissolved in water. Targeted LC–MS analyses were performed on a Q Exactive Orbitrap mass spectrometer (Thermo Scientific) coupled to a Vanquish UPLC system (Thermo Scientific). The Q Exactive operated in polarity-switching mode. A Sequant ZIC-pHILIC column (2.1 mm i.d. × 150 mm, particle size of 5 µm, Millipore Sigma) was used for separation of metabolites. A 2.1 × 20-mm guard column with the same packing material was used for protection of the analytical column. The flow rate was set at 150 μl min^−1^. Buffers consisted of 100% acetonitrile for mobile phase A, and 0.1% NH_4_OH/20 mM CH_3_COONH_4_ in water for mobile phase B. The chromatographic gradient ran from 85% to 30% A in 20 min followed by a wash with 30% A and re-equilibration at 85% A. The raw data was processed using El-MAVEN (v.0.12.0). Metabolites and their ^13^C isotopologues were identified on the basis of exact mass within 5 ppm and standard retention times. Natural isotope abundance across isotopologues was corrected using IsoCorrectoR before calculation of isotopologue distributions and percentage enrichment.

### Short-chain acyl-CoA analysis

Short-chain acyl-CoAs were extracted using pre-cooled 90% methanol from 10-cm dishes. Samples were centrifuged at 4 °C for 20 min at 20,000*g*. The supernatants containing short-chain acyl-CoAs were cleaned up with a Sep-Pak column and then dried down. Targeted LC–MS analyses were performed on a Q Exactive Orbitrap mass spectrometer (Thermo Scientific) coupled to a Vanquish UPLC system (Thermo Scientific). The Q Exactive operated in positive mode. An Imtakt Cadenza CD-C18 column (2.0 mm i.d. × 150 mm) was used to separate metabolites. The flow rate was set at 150 μl min^−1^. Buffers consisted of 100% methanol for mobile phase B, and 5 mM CH_3_COONH_4_ in water for mobile phase A. Gradient ran from 2% to 15% B in 5 min, changed to 95% B in 3 min, and followed by a wash with 95% B and re-equilibration at 85% B. The MS data were processed using XCalibur v.4.1 (Thermo Scientific). Metabolites were identified based on exact mass within 5 ppm and standard retention times. Relative metabolite quantitation was performed based on the peak height of each metabolite.

### Lipid profiling

Lipids were extracted from samples according to a published method^52^. The extract was dried down using a SpeedVac and then reconstituted using acetonitrile/isopropanol/water 65:30:5 before LC–MS analysis. Chromatographic separation was performed on a Vanquish UHPLC system with a Cadenza CD-C18 3-µm packing column (Imtakt, 2.1 mm i.d. × 150 mm) coupled to a Q Exactive Orbitrap mass spectrometer (Thermo Scientific) via an Ion Max ion source with a HESI II probe (Thermo Scientific). The mobile phase consisted of buffer A, 60% acetonitrile, 40% water, 10 mM ammonium formate with 0.1% formic acid; and buffer B, 90% isopropanol, 10% acetonitrile, 10 mM ammonium formate with 0.1% formic acid. The LC gradient was 0–1.5 min, 32% buffer B; 1.5–4 min, 32–45% buffer B; 4–5 min, 45–52% buffer B; 5–8 min, 52–58% buffer B; 8–11 min, 58–66% buffer B; 11–14 min, 66–70% buffer B; 14–18 min, 70–75% buffer B; 21–25 min, isocratic 97% buffer B; and 25–25.1 min 97–32% buffer B; followed by 5 min of re-equilibration of the column before the next run. The flow rate was 200 μl min^−1^. A data-dependent mass spectrometric acquisition method was used for lipid identification. In this method, each MS survey scan was followed by up to ten MS/MS scans performed on the most abundant ions. Data were acquired in positive and negative modes. The electrospray parameters were spray voltage 3.0 kV; heated capillary temperature 350 °C; HESI probe temperature 350 °C; sheath gas, 35 units; and auxiliary gas 10 units. For MS scans, the parameters were resolution, 70,000 (at m/z 200); automatic gain control target, 3e6; maximum injection time, 200 ms; and scan range, 250–1,800 m/z. For MS/MS scans, the parameters were resolution, 17,500 (at 200 m/z); automatic gain control target, 1e5 ions; maximum injection time, 75 ms; isolation window, 1 m/z; and normalized collision energy, stepped 20, 30 and 40. The LC–MS files were processed using MS-DIAL software v.5.1 for lipid identification and relative quantitation^53^.

### Cholesterol and cholesteryl ester quantification

Intracellular free cholesterol and total cholesterol levels were measured using the Cholesterol/Cholesterol Ester-Glo Assay (Promega, J3191). Following the indicated treatments or genetic manipulations, cells were trypsinized, and an equal number of cells (50,000–200,000 per condition) were collected for analysis. Following centrifugation, cell pellets were resuspended in 200 μl of Cholesterol Lysis Buffer. From each sample, 50 μl of lysate was transferred to individual wells of a 96-well plate and incubated at 37 °C for 30 min. Next, 50 μl of Cholesterol Detection Reagent, with or without esterase, was added to the wells to measure total cholesterol or free cholesterol, respectively. The plate was shaken for 1 min and then incubated at room temperature for 1 h. Luminescence was measured using a plate reader. A cholesterol standard curve was included in each assay for quantification. Cholesteryl ester levels were calculated by subtracting free cholesterol from total cholesterol.

### Steroid measurements

Cellular metabolites were extracted from approximately 5 × 10^6^ cells using 70% methanol, followed by the addition of three volumes of methyl tert-butyl ether. Samples were then dried in a vacuum concentrator. The dried cell extracts were resuspended in 50 µl of 50% methanol in H_2_O and centrifuged at 21,000*g* for 20 min. The resulting supernatants were subjected to LC–MS analysis using an Agilent liquid chromatography system coupled to an Agilent 6546 quadrupole time-of-flight mass spectrometer equipped with a Kinetex C18 column (100 Å, 1.7 μm, 2.1 × 100 mm; Phenomenex). The mobile phases consisted of solvent A (H_2_O containing 0.1% formic acid) and solvent B (methanol containing 0.1% formic acid). Samples (10 µl) were injected at a flow rate of 0.35 ml min^−1^, and the column was maintained at 40 °C. The LC gradient was 0–1 min, 99.5% A; 1–4 min, 99.5% to 2% A; 4–6 min, 2% A; 6–6.1 min, 2% to 99.5% A; and 6.1–7 min, 99.5% A.

Steroid peaks were identified by comparison with authentic standards. A fixed amount of D3-DHT was added to each sample before extraction as an internal standard to control for technical variation in extraction efficiency and instrument response. Calibration curves were generated using authentic steroid standards, and absolute analyte amounts were determined from analyte-to-internal standard peak area ratios. Final androgen levels were normalized to the number of cells used for metabolite extraction.

For ^13^C-cholesterol tracing experiments, tumour cells were cultured in medium containing charcoal-stripped serum for 3 days, followed by incubation with 20 μg ml^−1^ ^13^C-cholesterol (MedChemExpress, HY-N0322S5) for 2 days. Metabolites were then extracted as described above. Cholesterol-d_4_ (MedChemExpress, HY-N0322S6) was added to each sample as an internal standard to control for technical variation in extraction and instrument response. LC–MS analysis was used to detect labelled steroid products, and ^13^C-pregnenolone was assigned based on the expected isotopic mass shift and chromatographic behaviour relative to authentic standards. Relative labelling abundance was calculated from the corresponding peak area ratios after normalization to the internal standard^54^.

### Global gene expression analysis (RNA-seq)

RNA was extracted from cells using the Ambion PureLink RNA Mini kit (Life Technologies) according to manufacturer’s instructions and treated with DNase I (Amplification grade, Sigma-Aldrich). Total RNA integrity was checked using a 2100 Bioanalyzer (Agilent Technologies). RNA concentrations were measured using the NanoDrop system (Thermo Fisher Scientific). Preparation of RNA sample library and RNA-seq were performed by the Genomics Core Laboratory at Weill Cornell Medicine.

RNA was prepared using TruSeq Stranded mRNA Sample Library Preparation kit (Illumina), according to the manufacturer’s instructions. The normalized cDNA libraries were pooled and sequenced with paired-end 100 bp on NovaSeq 6000 sequencer.

The raw sequencing reads in BCL format were processed through bcl2fastq v.2.20 (Illumina) for FASTQ conversion and demultiplexing. After trimming the adaptors with cutadapt (v.1.18) (https://cutadapt.readthedocs.io/en/v1.18/), RNA reads were aligned and mapped to the GRCh38 human reference genome by STAR (v.2.5.2) (https://github.com/alexdobin/STAR)^55^, and transcriptome reconstruction was performed by Cufflinks (v.2.1.1) (http://cole-trapnell-lab.github.io/cufflinks/). The abundance of transcripts was measured with Cufflinks in fragments per kilobase of exon model per million mapped reads (FPKM)^56,57^. Raw read counts per gene were extracted using HTSeq-count (v.0.11.2)^58^. Gene expression profiles were constructed for differential expression, clustering and principal-component analyses with the DESeq2 package (https://bioconductor.org/packages/release/bioc/html/DESeq2.html)^59^. For differential expression analysis, pairwise comparisons between groups were assessed using parametric tests where read counts follow a negative binomial distribution with a gene-specific dispersion parameter. Corrected *P* values were calculated based on the Benjamini–Hochberg method to adjust for multiple testing.

### Published data reanalysis

Gene expression profiling data from tumour tissues of patients with PCa were retrieved from two previously published studies and reanalysed (Extended Data Figs. 1a and 2f)^19,60^. Genes involved in the BCAA catabolism pathway were compared between primary and metastatic tumour tissues. The original gene expression data were obtained from the Gene Expression Omnibus under accession number GSE32269.

Metabolomics data from tumour tissues of patients with PCa were collected from a previous publication and reanalysed by Benedetti et al.^61^. (Extended Data Figs. 1b,c and 3i). The levels of PLC and cholesterol were analysed and compared among patients with varying Gleason scores.

Additionally, metabolomics data from PCa patient-derived xenografts were extracted from a previous study^29^ (Extended Data Fig. 6k). PLC levels were analysed to assess the impact of castration and castration resistance on its level.

### Data analysis and statistics

No statistical methods were used to predetermine sample sizes. Sample sizes were selected based on previous experience and were similar to those reported in previous studies using comparable experimental models and methods. Data collection and analysis were not performed blindly to the conditions of the experiments. No animals or data points were excluded from the analyses. Data analyses were performed using Microsoft Excel 2013 and GraphPad Prism v.8.2.1. Statistical significance was determined using two-tailed Student’s *t*-test, one-way analysis of variance (ANOVA) or two-way ANOVA. For the metabolomics analysis and RNA-seq analysis, *P* values for each comparison were adjusted using the Benjamini–Hochberg procedure using a false discovery rate of 20%. In all analyses, values of *P* < 0.05 were considered significant. Data are presented as the mean ± s.d. of at least three independent samples. The number of independent samples and the statistical methods used in each experiment are specified in the figure legends. Data distribution was assumed to be normal but was not formally tested. For DAVID functional annotation analyses of RNA-seq experiments, the EASE score, a modified Fisher exact *P* value, was used to determine significance, as recommended.

### Reporting summary

Further information on research design is available in the Nature Portfolio Reporting Summary linked to this article.

## Supplementary information

Reporting Summary

Supplementary Table 1 Lipid profiling.

Supplementary Table 2 Diet formulation.

Supplementary Table 3 Antibodies.

Supplementary Table 4 Primers.

Supplementary Table 5 shRNA and siRNA.

## Source data

Source Data Fig. 1 Statistical source data.

Source Data Fig. 1 Unprocessed western blots.

Source Data Fig. 2 Statistical source data.

Source Data Fig. 2 Unprocessed western blots.

Source Data Fig. 3 Statistical source data.

Source Data Fig. 4 Statistical source data.

Source Data Fig. 4 Unprocessed western blots.

Source Data Fig. 5 Statistical source data.

Source Data Fig. 5 Unprocessed western blots.

Source Data Fig. 6 Statistical source data.

Source Data Fig. 6 Unprocessed western blots.

Source Data Extended Data Fig. 1 Statistical source data.

Source Data Extended Data Fig. 1 Unprocessed western blots.

Source Data Extended Data Fig. 2 Statistical source data.

Source Data Extended Data Fig. 2 Unprocessed western blots.

Source Data Extended Data Fig. 3 Statistical source data.

Source Data Extended Data Fig. 3 Unprocessed western blots.

Source Data Extended Data Fig. 4 Statistical source data.

Source Data Extended Data Fig. 4 Unprocessed western blots.

Source Data Extended Data Fig. 5 Statistical source data.

Source Data Extended Data Fig. 5 Unprocessed western blots.

Source Data Extended Data Fig. 6 Statistical source data.

Source Data Extended Data Fig. 6 Unprocessed western blots.

Source Data Extended Data Fig. 7 Statistical source data.

Source Data Extended Data Fig. 7 Unprocessed western blots.

## Data Availability

All data generated or analysed during this study are included in this article and its Supplementary Information, or are available in publicly accessible repositories as described in Methods. RNA sequencing data have been deposited in the Gene Expression Omnibus under accession code GSE272086. Raw metabolomics data have been deposited in the Zenodo repository at https://doi.org/10.5281/zenodo.21047051 (ref. ^62^). Source data are provided with this paper.

## Code availability

No custom computational code or algorithms were developed for this study. Data analyses were performed using standard, commercially available or publicly available software as described in Methods.
