# Supplementary material for: Isoleucine and valine promote prostate cancer progression via propionyl-CoA-mediated cholesterol metabolism
Source: Nat Metab. 2026 Aug 20;8(8):1772–90. doi: 10.1038/s42255-026-01583-z (PMC13493318; doi:10.1038/s42255-026-01583-z)
Supplement: Supplementary file 2 [file 42255_2026_1583_MOESM13_ESM.pdf]

Figure 4a

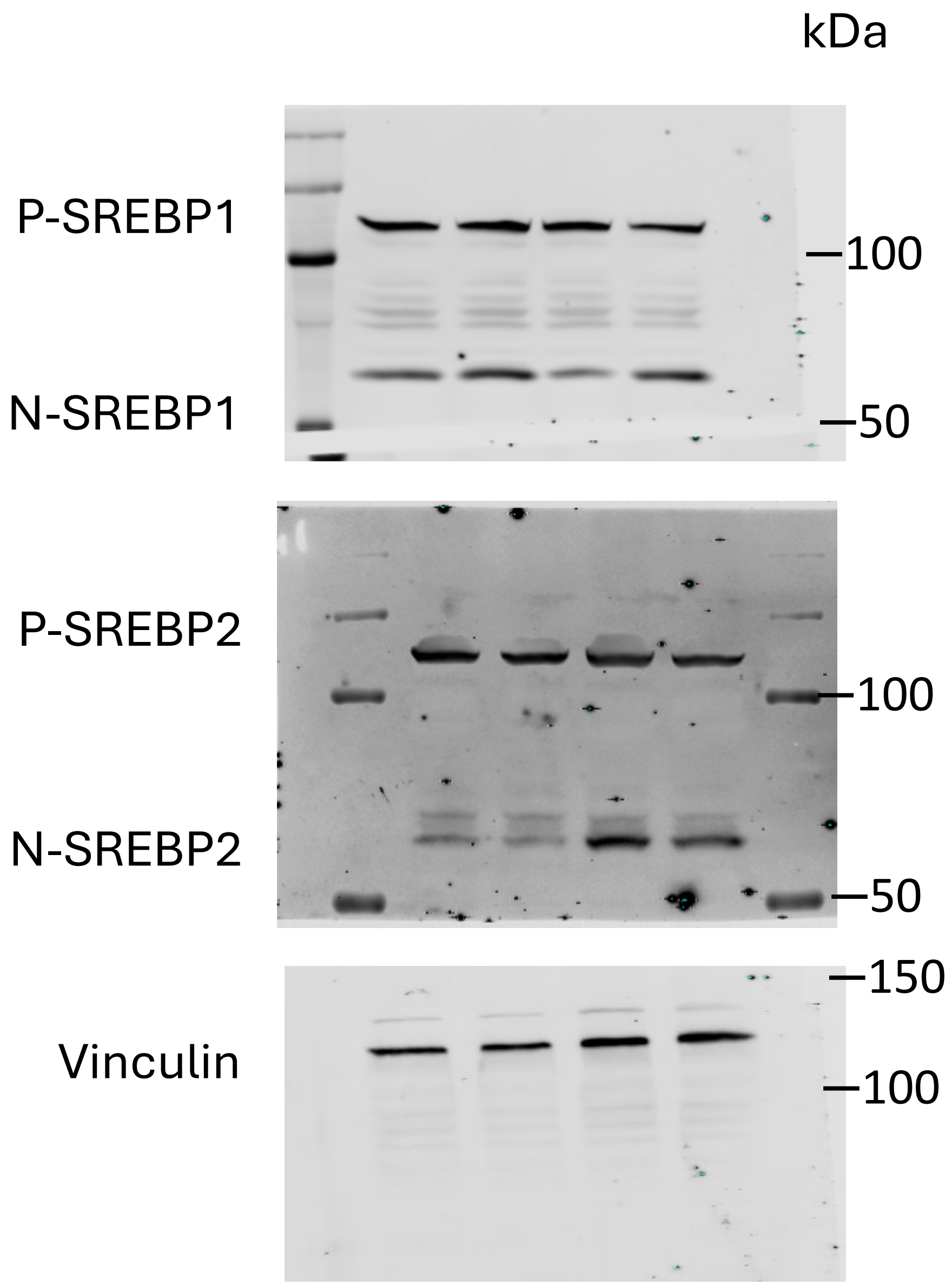
kDa
P-SREBP1
N-SREBP1
100
50
P-SREBP2
N-SREBP2
100
50
150
Vinculin
100

Figure 4b

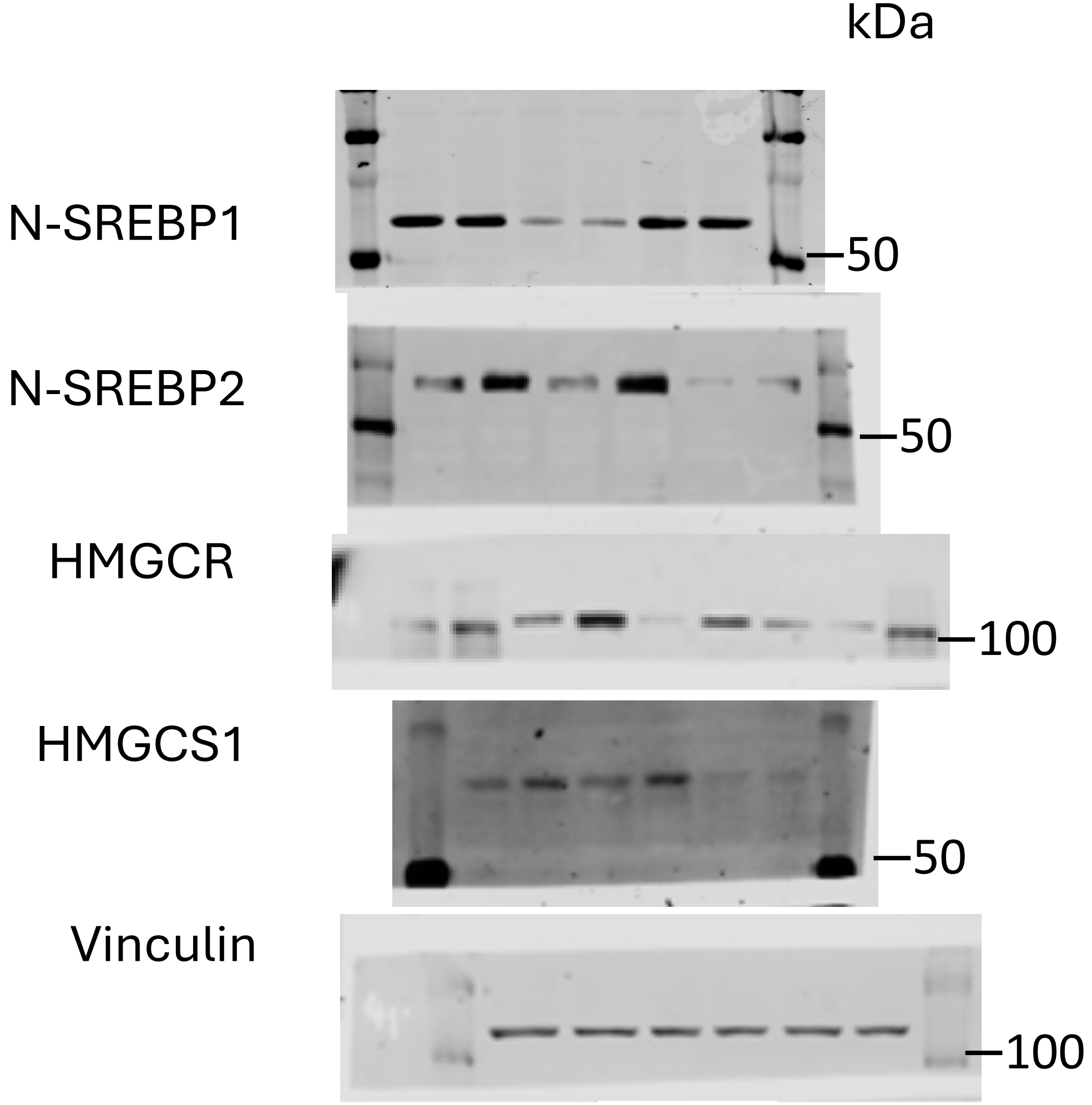

Figure 4d

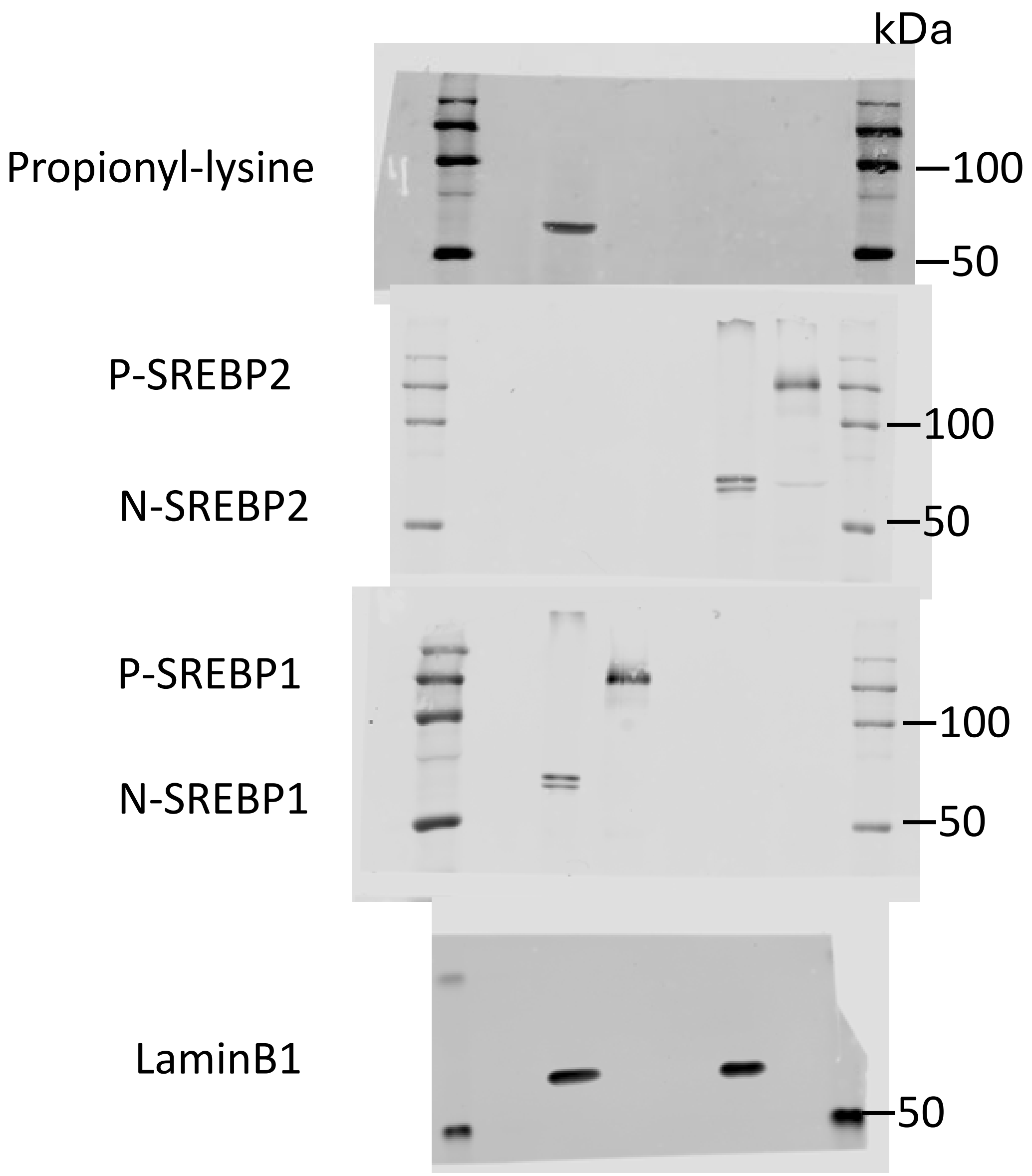
kDa
Propionyl-lysine
100
50
P-SREBP2
100
N-SREBP2
50
P-SREBP1
100
N-SREBP1
50
LaminB1
50

Figure 4e

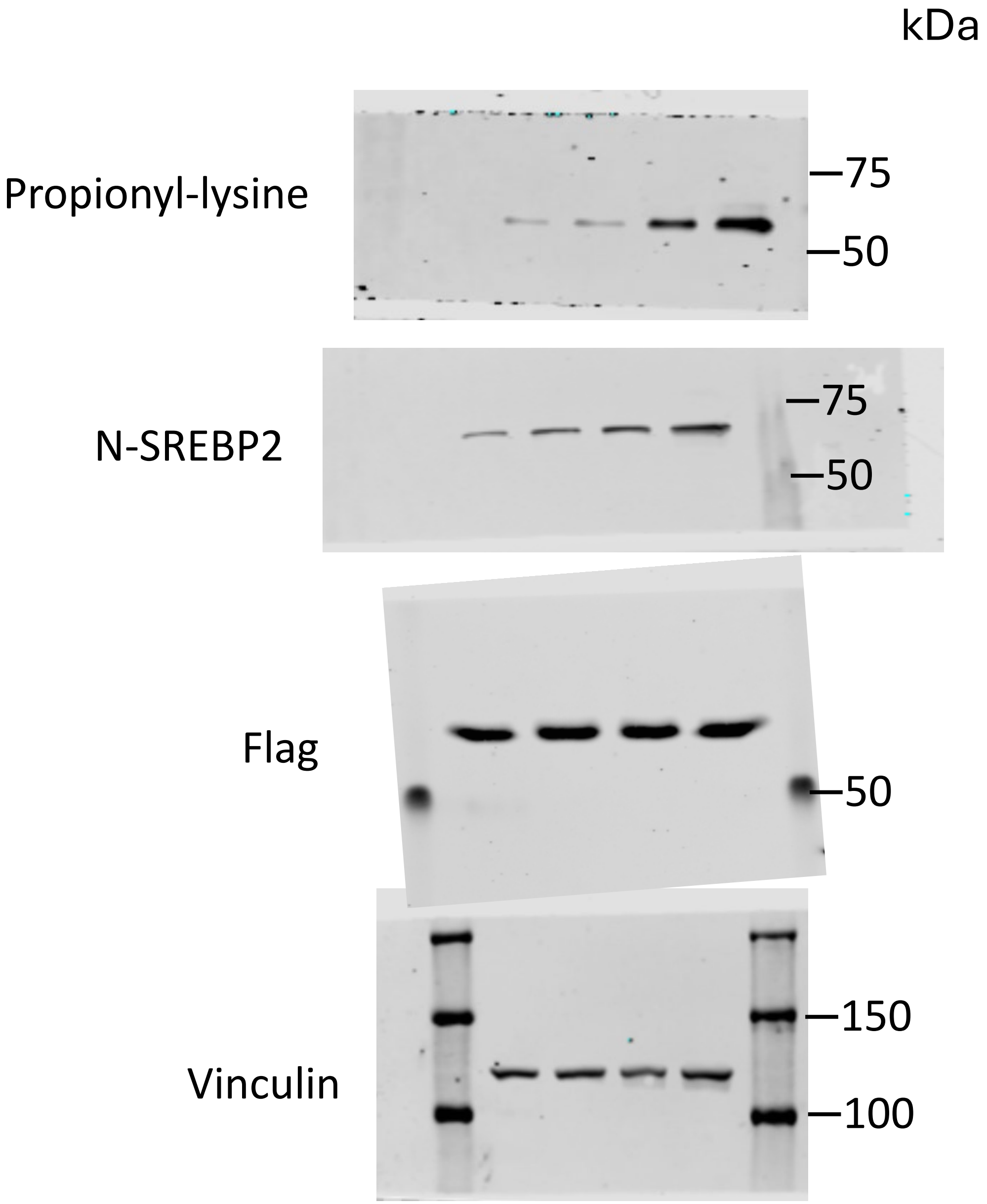

kDa
Propionyl-lysine
75
50
N-SREBP2
75
50
Flag
50
Vinculin
150
100

Figure 4f

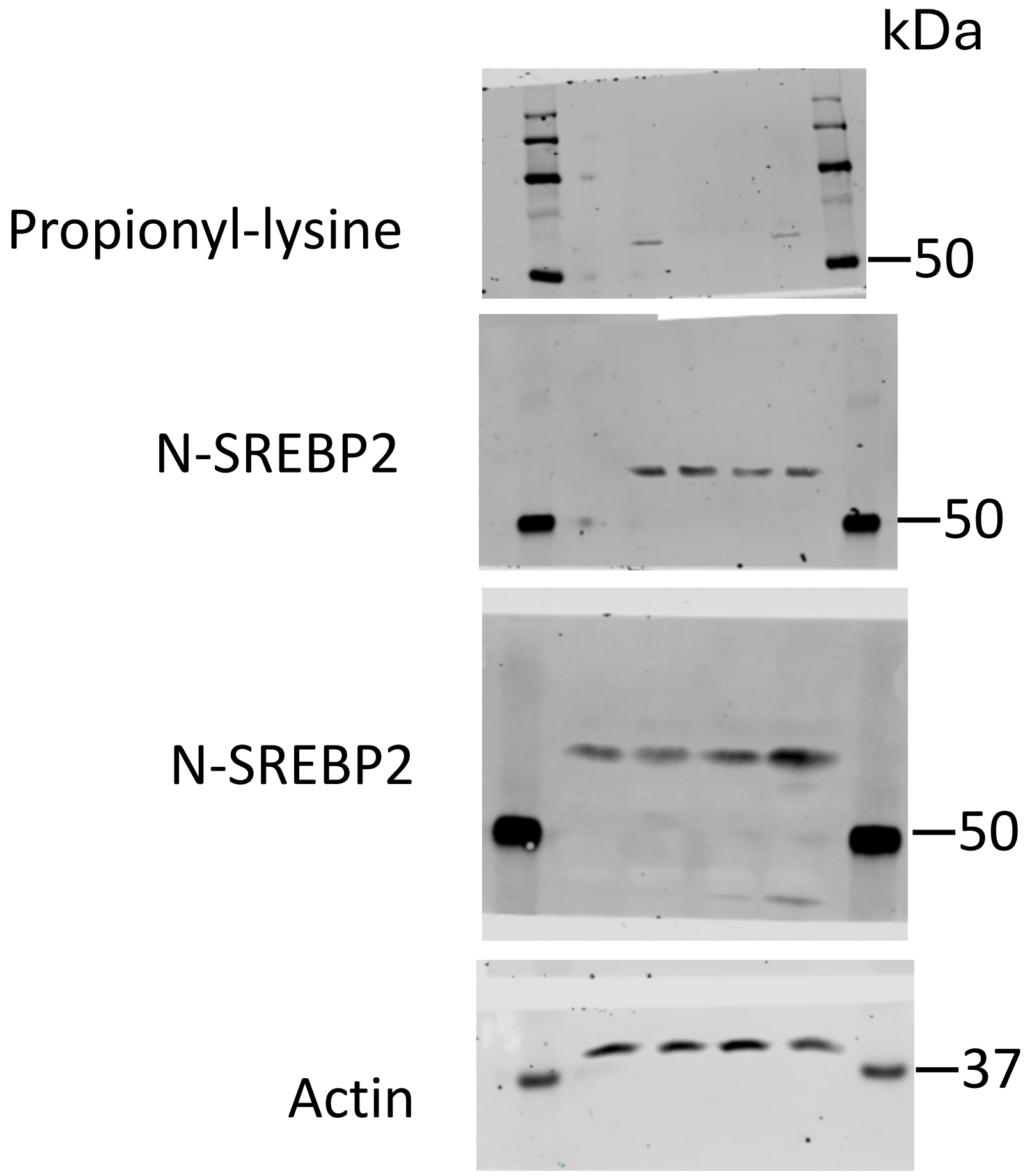

Figure 4g

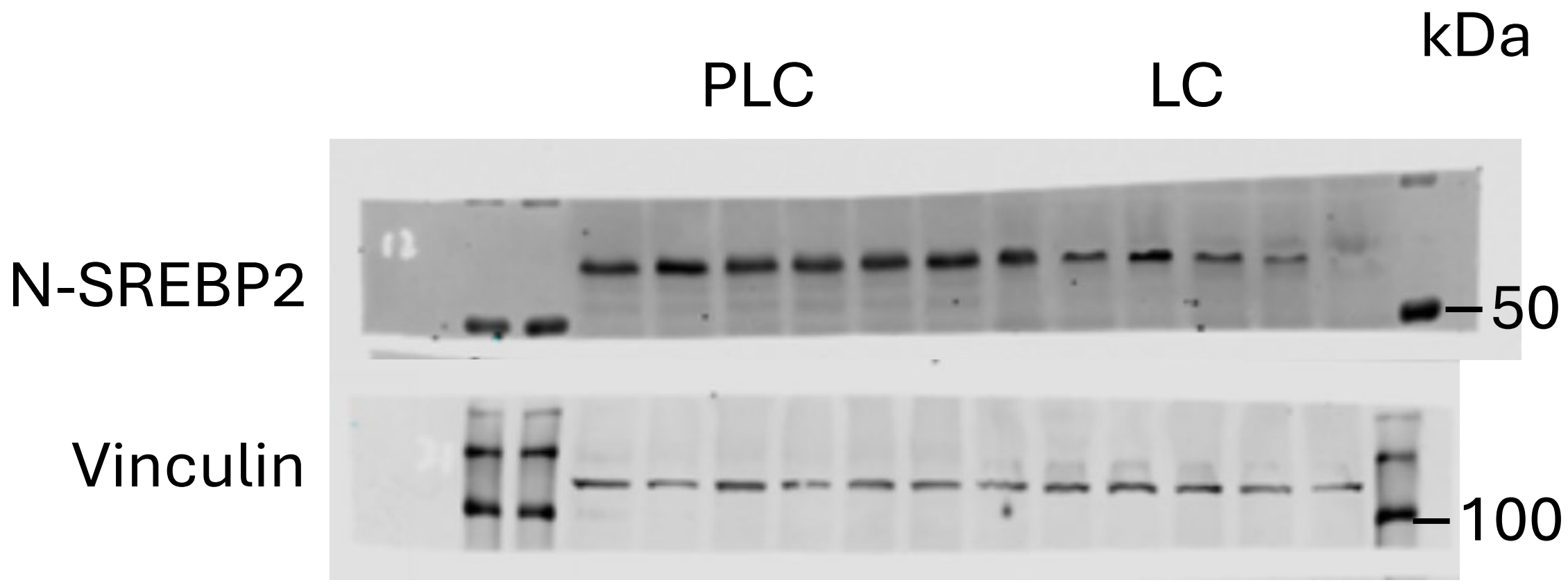

Figure 4h

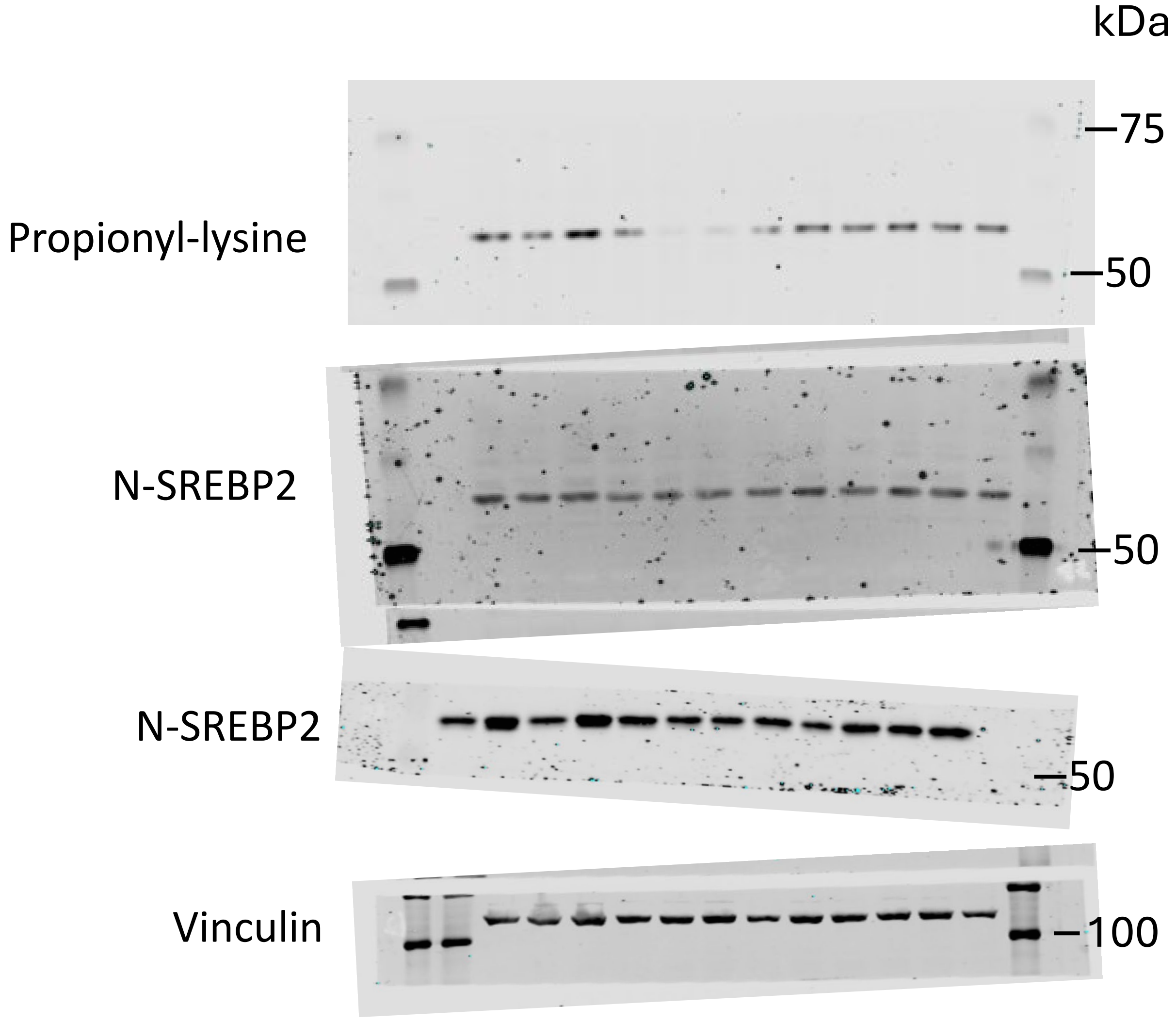
kDa
Propionyl-lysine
75
50
N-SREBP2
50
N-SREBP2
50
Vinculin
100

Figure 4i

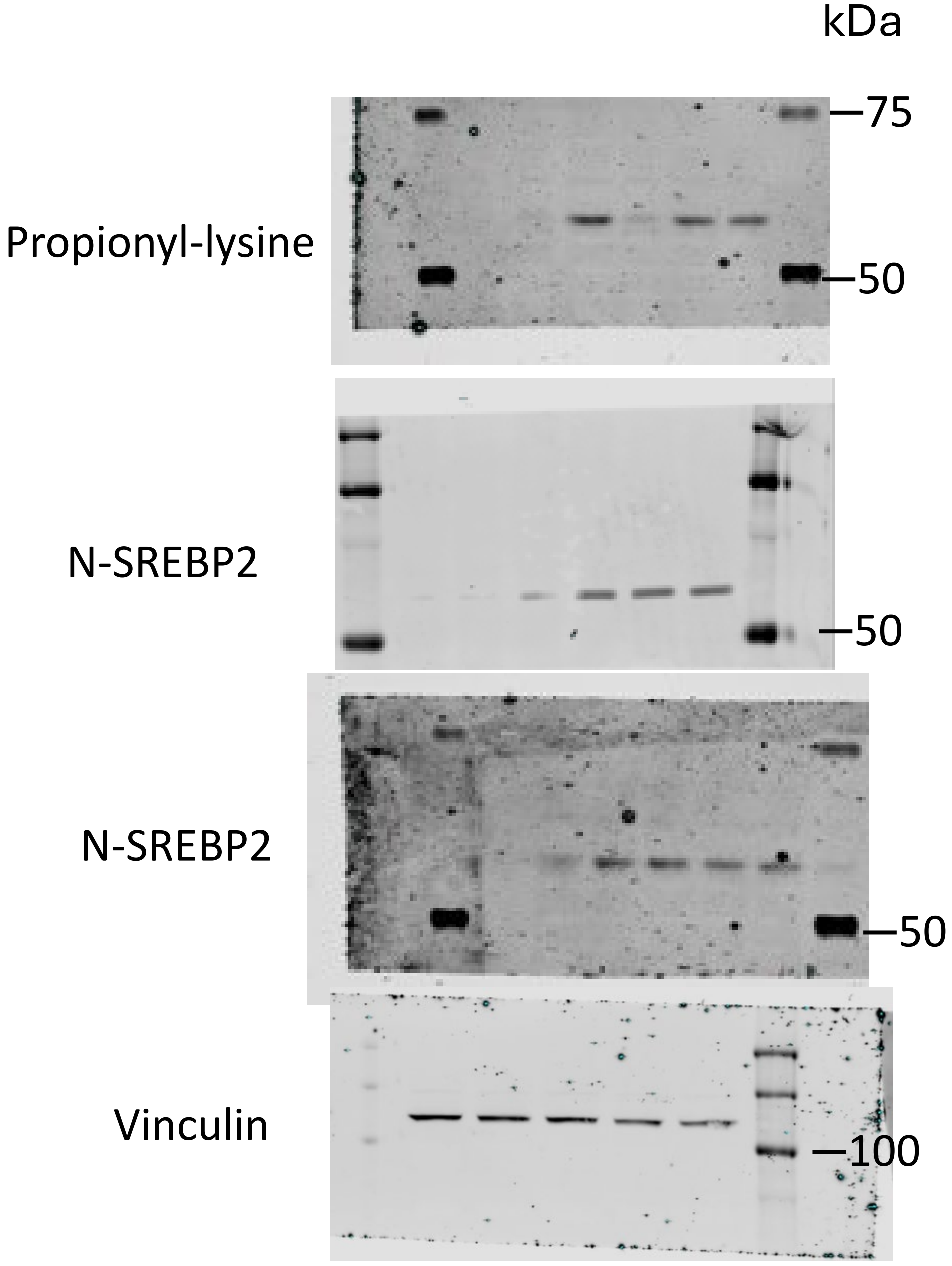
kDa
Propionyl-lysine
75
50
N-SREBP2
50
N-SREBP2
50
Vinculin
100
