# Supplementary material for: Isoleucine and valine promote prostate cancer progression via propionyl-CoA-mediated cholesterol metabolism
Source: Nat Metab. 2026 Aug 20;8(8):1772–90. doi: 10.1038/s42255-026-01583-z (PMC13493318; doi:10.1038/s42255-026-01583-z)

Figure 5f

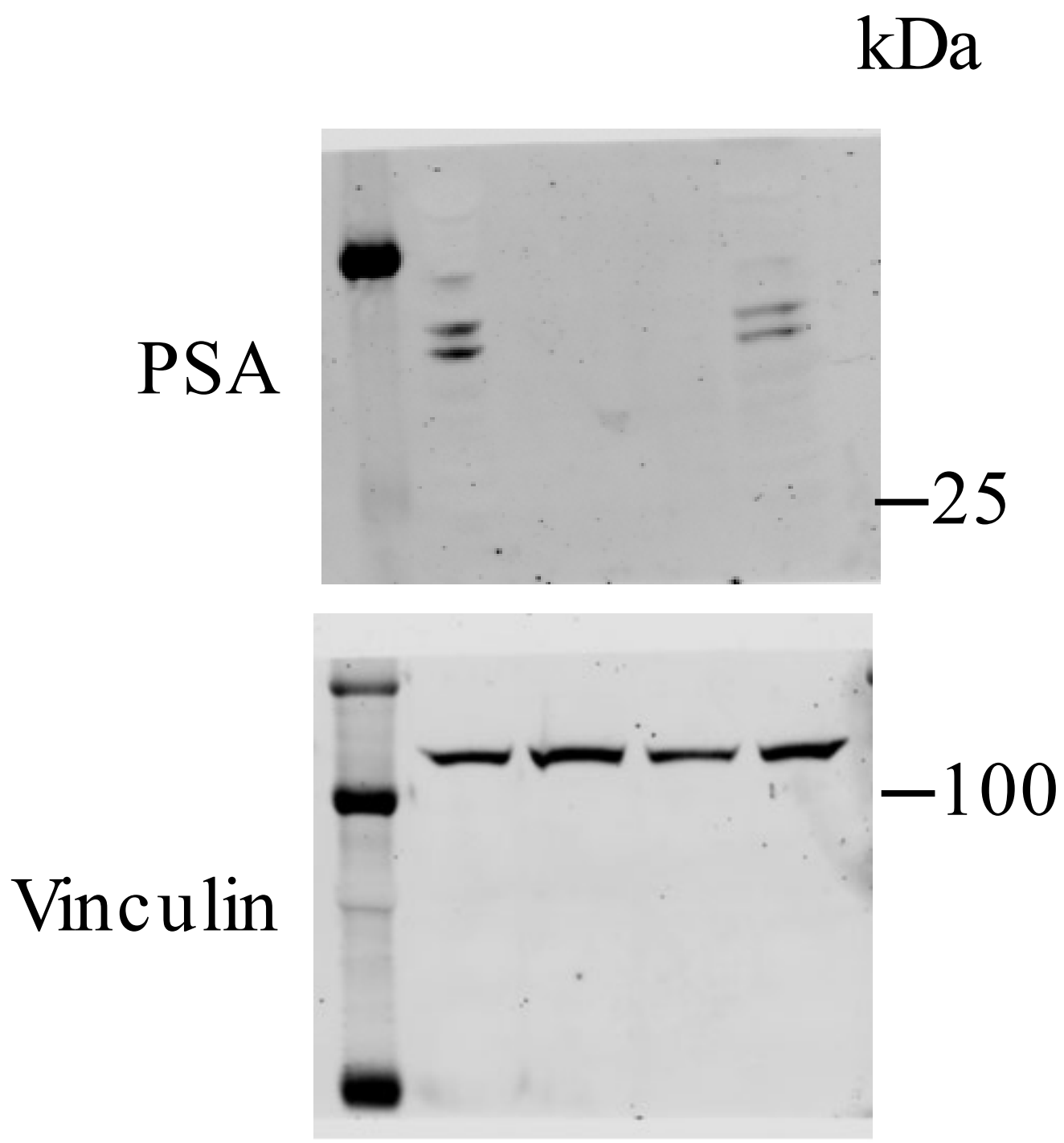

Supplement: Supplementary file 3 [file 42255_2026_1583_MOESM15_ESM.pdf]
