# Supplementary material for: Isoleucine and valine promote prostate cancer progression via propionyl-CoA-mediated cholesterol metabolism
Source: Nat Metab. 2026 Aug 20;8(8):1772–90. doi: 10.1038/s42255-026-01583-z (PMC13493318; doi:10.1038/s42255-026-01583-z)
Supplement: Supplementary file 6 [file 42255_2026_1583_MOESM1_ESM.pdf]

nature portfolio

Corresponding author(s): John Blenis

Last updated by author(s): Jun 25, 2026

Reporting Summary

Nature Portfolio wishes to improve the reproducibility of the work that we publish. This form provides structure for consistency and transparency in reporting. For further information on Nature Portfolio policies, see our Editorial Policies and the Editorial Policy Checklist.

Statistics

For all statistical analyses, confirm that the following items are present in the figure legend, table legend, main text, or Methods section.

n/a	Confirmed	
☐	☒	The exact sample size (*n*) for each experimental group/condition, given as a discrete number and unit of measurement
☐	☒	A statement on whether measurements were taken from distinct samples or whether the same sample was measured repeatedly
☐	☒	The statistical test(s) used AND whether they are one- or two-sided<br>*Only common tests should be described solely by name; describe more complex techniques in the Methods section.*
☐	☒	A description of all covariates tested
☐	☒	A description of any assumptions or corrections, such as tests of normality and adjustment for multiple comparisons
☐	☒	A full description of the statistical parameters including central tendency (e.g. means) or other basic estimates (e.g. regression coefficient) AND variation (e.g. standard deviation) or associated estimates of uncertainty (e.g. confidence intervals)
☐	☒	For null hypothesis testing, the test statistic (e.g. *F*, *t*, *r*) with confidence intervals, effect sizes, degrees of freedom and *P* value noted<br>*Give P values as exact values whenever suitable.*
☒	☐	For Bayesian analysis, information on the choice of priors and Markov chain Monte Carlo settings
☒	☐	For hierarchical and complex designs, identification of the appropriate level for tests and full reporting of outcomes
☒	☐	Estimates of effect sizes (e.g. Cohen's *d*, Pearson's *r*), indicating how they were calculated

Our web collection on statistics for biologists contains articles on many of the points above.

Software and code

Policy information about availability of computer code

Data collection	Image J Fiji 2.9.0; LI-COR Odyssey CLx Imaging System; Perkin Elmer EnVision 2102-0010 Multilabel Microplate Reader; QuantStudio 6 Real-Time PCR System; the metabolite sample was analyzed using an Agilent 1290 LC system coupled to an Agilent 6530 quadrupole time-of-flight (QTOF) mass spectrometer equipped with a 5 µm, 4.6 mm × 50 mm UPLC C4 column (Bio-Bond).
Data analysis	Microsoft Excel and Prism - GraphPad 9 were used for statistic analysis; for RNA seq, raw read counts per gene were extracted using HTSeq-count v0.11.2 9. Gene expression profiles were constructed for differential expression, cluster, and principle component analyses with the DESeq2 package; The MS data was processed using XCalibur 4.1 (Thermo Scientific) to obtain the metabolite signal intensities; The LC-MS files were processed using MS-DIAL software version 5.1 for lipid identification and relative quantitation; Real time q-PCR was analyzed by QuantStudio Real-Time PCR Software v1.3 (Life Technologies);

For manuscripts utilizing custom algorithms or software that are central to the research but not yet described in published literature, software must be made available to editors and reviewers. We strongly encourage code deposition in a community repository (e.g. GitHub). See the Nature Portfolio guidelines for submitting code & software for further information.

Data

Policy information about availability of data

All manuscripts must include a data availability statement. This statement should provide the following information, where applicable:

- Accession codes, unique identifiers, or web links for publicly available datasets
- A description of any restrictions on data availability
- For clinical datasets or third party data, please ensure that the statement adheres to our policy

All data generated or analyzed during this study are included in this published paper under the Source Data file, or has been previously published as described in the Methods. The RNA-seq data generated in this study have been deposited in the Gene Expression Omnibus (GEO) repository under accession code GSE272086. The raw data for the metabolomics were deposited in MetaboLights with identifier: MTBLS11071. All the other data are available within the paper and its Supplementary Information.

Research involving human participants, their data, or biological material

Policy information about studies with human participants or human data. See also policy information about sex, gender (identity/presentation), and sexual orientation and race, ethnicity and racism.

Reporting on sex and gender	N/A
Reporting on race, ethnicity, or other socially relevant groupings	N/A
Population characteristics	N/A
Recruitment	N/A
Ethics oversight	N/A

Note that full information on the approval of the study protocol must also be provided in the manuscript.

Field-specific reporting

Please select the one below that is the best fit for your research. If you are not sure, read the appropriate sections before making your selection.

☒ Life sciences ☐ Behavioural & social sciences ☐ Ecological, evolutionary & environmental sciences

For a reference copy of the document with all sections, see nature.com/documents/nr-reporting-summary-flat.pdf

Life sciences study design

All studies must disclose on these points even when the disclosure is negative.

Sample size	The sample sizes for both cell culture and mouse experiments were determined based on preliminary experiments and power analyses to ensure sufficient statistical power to detect significant differences between groups. For cell culture experiments, a minimum of three biological replicates was used per condition to ensure reproducibility and reduce variability. For mouse experiments, sample sizes were calculated using G*Power software, assuming an effect size of 0.8, a significance level (α) of 0.05, and a power (1-β) of 0.8, leading to a group size of at least 6 animals. These sizes were chosen based on similar studies published in the field to ensure reliability and consistency with accepted practices.
Data exclusions	Data exclusion criteria were pre-established before the experiments to avoid bias. For cell culture experiments, samples were excluded if contamination was detected. For mouse studies, animals showing signs of unrelated illness or physical injury that could influence the study outcomes were excluded.
Replication	All experiments were independently repeated at least three times with biological replicates. Key findings were validated using alternative methodologies where applicable (e.g., qPCR and western blot for molecular markers in cell culture experiments). RNA-seq, lipidomics analyses were only performed once with three biological replicates.
Randomization	For cell culture experiments, samples were randomly assigned to treatment groups; in mouse experiments, animals were randomly assigned to experimental groups at the start of the study to ensure even distribution of baseline characteristics
Blinding	The IHC staining and tumor tissue oil Red O staining were performed by the core facility, the sample groups were binded to them. For the other experiment, blinding was not implemented because the experimental procedures (e.g., drug administration, sample preparation, data acquisition) and outcomes (e.g., molecular marker expression, tumor volume) were objective and quantifiable, minimizing the risk of bias. Since data acquisition involved automated equipment with preset parameters (e.g., plate reader, imaging software), subjective interpretation was avoided.

Reporting for specific materials, systems and methods

We require information from authors about some types of materials, experimental systems and methods used in many studies. Here, indicate whether each material, system or method listed is relevant to your study. If you are not sure if a list item applies to your research, read the appropriate section before selecting a response.

Materials & experimental systems

n/a	Involved in the study
☐	☒ Antibodies
☐	☒ Eukaryotic cell lines
☒	☐ Palaeontology and archaeology
☐	☒ Animals and other organisms
☒	☐ Clinical data
☒	☐ Dual use research of concern
☒	☐ Plants

Methods

n/a	Involved in the study
☒	☐ ChIP-seq
☒	☐ Flow cytometry
☒	☐ MRI-based neuroimaging

Antibodies

Antibodies used

The antibodies used were: Perilipin-2 (95109, Cell Signaling Technology, 1:1000), PCCA (ab187686, Abcam, 1:2000), ACAT1 (44276, Cell Signaling Technology, 1:2000), ALDH6A1 (365160, Santa Cruz, 1:1000), HMGCR (153331, Thermo Fisher Scientific, 1:1000), HMGCS1 (ab87246, Abcam, 1:1000), SREBP-1 (sc13551, Santa Cruz, 1:500), SREBP-2 (ab30682, Abcam, 1:1000), PCNA (13110, Cell Signaling Technology, 1:2000), MCM2 (3619, Cell Signaling Technology, 1:1000), Pan anti-propionyl lysine (PTM201, PTM Biolabs, 1:500), PSA (2475, Cell Signaling Technology, 1:1000), phosphor-S6K (Thr389) (9234, Cell Signaling Technology, 1:1000), phosphor-S6 (Ser235/236) (4858, Cell Signaling Technology, 1:1000), phosphor-S6 (Ser240/244) (5364, Cell Signaling Technology, 1:1000), Ubiquitin (P37) (58395, Cell Signaling Technology, 1:1000) and Vinculin (V9264, Sigma-Aldrich, 1:5000). Following primary antibody incubation, membranes were incubated with donkey anti-rabbit Alexa Fluor 488 (A-21206, Thermo Fisher Scientific, 1:10,000) or donkey anti-mouse Alexa Fluor 555 (A31570, Thermo Fisher Scientific, 1:10,000) secondary antibodies.

Validation

All antibodies utilized in this study were commercially sourced, with validation details accessible on the vendor's website using the respective product numbers.

Eukaryotic cell lines

Policy information about cell lines and Sex and Gender in Research

Cell line source(s)

LNCaP (CRL-1740), C4-2 (CRL-3314), C4-2B (CRL-3315), 22Rv1 (CRL-2505), PC3 (CRL-1435), and DU145 (HTB-81) prostate cancer cell lines were purchased from ATCC. HEK293T cells were obtained from GenHunter (Q401).

Authentication

All cell lines used in this study were commercially obtained from reputable vendors, who also performed their authentication. Short tandem repeat (STR) profiling was used and the resulting profiles were compared with reference profiles in established cell-line databases.

Mycoplasma contamination

Routine testing confirmed all cell lines were mycoplasma-free.

Commonly misidentified lines
(See ICLAC register)

N/A

Animals and other research organisms

Policy information about studies involving animals; ARRIVE guidelines recommended for reporting animal research, and Sex and Gender in Research

Laboratory animals

Male nu/nu athymic mice (Envigo), aged 7–8 weeks

Wild animals

N/A

Reporting on sex

The disease in this study is prostate cancer, therefore only male mice were used in the study

Field-collected samples

N/A

Ethics oversight

All procedures were carried out in compliance with Weill Cornell Medicine Institutional Animal Care and Use Committee (IACUC) protocols.

Note that full information on the approval of the study protocol must also be provided in the manuscript.

Plants

Seed stocks	N/A
Novel plant genotypes	N/A
Authentication	N/A
